# DRIM modulates Src activation and regulates angiogenic functions in vascular endothelial cells

**DOI:** 10.1002/cbin.12265

**Published:** 2024-12-08

**Authors:** Jia Tong, Xuefei Dong, Tracey A. Martin, Yiming Yang, Bo Dong, Wen G. Jiang

**Affiliations:** ^1^ Department of Geriatric Medicine, Shandong Provincial Hospital Affiliated to Shandong First Medical University Shandong First Medical University Jinan China; ^2^ Cardiff China Medical Research Collaborative Division of Cancer and Genetics, Cardiff University School of Medicine Cardiff UK; ^3^ Department of Cardiology, Shandong Provincial Hospital, Cheeloo College of Medicine Shandong University Jinan Shandong China

**Keywords:** DRIM, FAK, phosphorylation, Src, UTP20, vascular endothelial cell

## Abstract

Downregulated in Metastasis Protein (DRIM) was discovered in malignant epithelial cells and was thought to be mainly a nucleus protein affecting cancer cells. Recent single‐cell sequencing analysis suggests that DRIM is abundantly expressed in vascular endothelial cells. There has been no knowledge of the role of DRIM in the endothelium. In the present study, using protein fraction method and cell imaging, we identified that the DRIM protein was abundantly present in both nucleus and the cytoskeletal fractions of human vascular endothelial cells. Knockdown of DRIM in the endothelial cells significantly affected growth, migration, and angiogenic tubule formation. Proteomics analyses revealed that Src was an important direct target protein of DRIM, a finding further confirmed by protein interaction assay. Silencing DRIM activated the tyrosine 419 site phosphorylation of Src kinase in endothelial cells, thereby affecting the downstream proteins of Src including p‐FAK and p‐STAT3, and exerting biological effects. To conclude, our results provide evidence of DRIM being a nuclear and cytoskeletal‐associated protein, having a novel key role of the protein in vascular endothelial cells.

## 1 INTRODUCTION

The endothelium is the single cell layer lining blood vascular endothelial cells or vascular endothelial cells, forming a semi‐selective barrier to separate blood flow from peripheral organs and tissues and playing a crucial role in the homeostasis of the vascular system throughout the whole body. Endothelial cell growth is regulated by various signaling molecules, such as vascular endothelial growth factor (VEGF), basic fibroblast growth factor (bFGF), and platelet‐derived growth factor (PDGF). VEGF, bFGF, and PDGF initiate signaling pathways by activating their respective receptors (VEGFR, FGFR, and PDGFR) (Ferrara, 2000; Carmeliet et al., 2001). The phosphorylation state of these receptors recruits and activates Src family kinases, which stimulates various downstream signaling pathways and ultimately leads to biological effects like cell proliferation, migration, and angiogenesis (Sandilands et al.,^2011^; Saraswati & Agrawal, 2013; Sun et al., 2010; Tzeng et al., 2015; Xie et al., 2021). Migration of endothelial cells is a key aspect in embryogenesis, tissue regeneration, wound healing, and angiogenesis in cancer metastasis (Dass & Choong, 2008; Gariano & Gardner, 2005; Lamalice et al., 2007; Marmé, 2018). Angiogenesis is a fundamental process with widespread implications in wound healing, cancer research, cardiovascular diseases, ophthalmic diseases, and regenerative medicine (Fontana et al., 2015; Maishi & Hida, 2017; Montalescot et al., 2013). Being able to understand and manipulate this process has the potential to significantly improve treatments and outcomes across these diverse medical fields.

Downregulated in Metastasis Protein (DRIM) is also known as 1A6/DRIM or UTP20 Small Subunit Processome Component. DRIM was first described as a molecule downregulated in metastasis in a study with submodels of a breast cancer cell line MDA MB‐435, in which the highly metastatic subline 4A4 had little or no expression of DRIM whereas the non‐metastatic subline 2C5 overexpressed DRIM (Schwirzke et al., 1998). However, subsequent studies have shown that the link between DRIM and the metastatic potential of breast cancer cells was not clear and that the initial finding was due to a genetic variation of one of the sublines (Goodison et al., 2003). The coding gene of DRIM, located to human chromosome 12q23 ~ 24 was identical to another gene, namely UTP20 (Goodison et al., 2003; Wang et al., 2007), one of the components of the U3 small nucleolar RNA complex. Peng et al. have reported that knockdown of DRIM caused p53 activation and inhibited cell proliferation by arresting cells at G1 stage in U2OS cell (Peng et al., 2010). DRIM was thought to relate to the metastatic potential and act as a putative nuclear protein regulating certain gene expression in some cancer cells, and that transcription expression in cancer cells is activated by a transcription factor USF2 (Xing et al., 2005). From the early discovery, DRIM protein has always been indicated as a nucleus protein (Goodison et al., 2003; Liu et al., 2006). In addition to the nucleus, there is a tentative suggestion that the DRIM protein may also be present elsewhere in the cells (Huang et al., 2004). Although recent single‐cell sequencing analysis suggests that DRIM is abundantly expressed in vascular endothelial cells, the expression, the cellular location, and its overall biological role, along with potential biochemical interaction partners in vascular endothelial cells, remain unknown.

In the present study, we investigated the biological functions, the cellular location, and the biochemical implications of DRIM in human vascular endothelial cells. We firstly demonstrated that the presence of DRIM in the cytoskeletal protein fraction as well as in the nucleus fractions (nuclear soluble and chromatin bound), and DRIM was associated with the tyrosine kinase Src signaling pathways and participated in the biological functions of vascular endothelial cells. Consequently, these findings provide a new perspective on understanding the comprehensive functions of DRIM and may reveal its potential roles and therapeutic targets in angiogenesis‐related diseases.

## 2 METHODS AND MATERIALS

### 2.1 Antibodies and reagents

The following antibodies (Ab) were used: anti‐DRIM (ab 86093, Abcam, Cambridge, England, UK), anti‐DRIM (sc109264, Santa Cruz Biotechnology, Santa Cruz, CA, USA), anti‐phospho‐Src (Tyr419) (ab4816, Abcam), anti‐phospho‐FAK(Tyr397) (ab81298, Abcam, Cambridge, UK), anti‐phospho‐STAT3 (Tyr705) (ab 76315, Abcam), anti‐FAK (ab131435, Abcam, Cambridge, UK), and anti‐Src (ab47405, Abcam). Anti‐HSP90 (SC‐69703) and anti‐vimentin (SC‐73614) was from Santa Cruz Biotechnologies. AZM475271 was purchased from Sigma‐Aldrich (SML2572, Dorset, England, UK). Fluorescence and HRP conjugated secondary antibodies and non‐immunized IgG were also obtained from Sigma‐Aldrich. Anti‐DRIM shRNA lentivirus was obtained from Santa Cruz Biotechnologies.

### 2.2 Cell lines and cell transfection

Human vascular endothelial cells (HECV) were purchased from Interlab (Interlab) and cultured in DMEM/F12 (Sigma‐Aldrich) supplemented with 10% foetal calf serum (FCS) (Sigma‐Aldrich) and 1% antibiotics (Sigma‐Aldrich). Immortalized human cerebral endothelial cell line, hCMEC/D3 (a kind gift from Dr Guilaume, Flury of Institute COCHIN) was cultured in endothelial cell growth medium (Sigma‐Aldrich), 5% FCS and 1% antibiotics.

Endothelial cells were transfected with DRIM shRNA lentivirus (Santa Cruz Biotechnologies) following the manufacturer's protocol. After incubation for the indicated time, cells were harvested for expression analysis and further experiments. All the cells were cultured in a humidified incubator at 37°C, 5% CO_2_, and 95% humidity.

### 2.3 Immunofluorescence (IFC)

Cells were seeded in a glass chamber slide and allowed to reach 50%–70% confluence. They were then fixed with 4% paraformaldehyde for 15 min. Then cells were permeabilized with 0.1% Triton X100 for 5 min, followed by brief washing with PBC buffer and blocking with 8% horse serum for 2 h. Primary antibodies (1:100 dilution) were added and the slide and kept in a 4°C refrigerator overnight, followed by extensive washing. Secondary antibodies, conjugated with either FITC or TRITC together with DAPI (4,6‐diamidino‐2‐phenylindole) (1:1000 dilution) were added to the respective wells for a further 2 h in the dark. The slides were then mounted using FluorSave^TM^ (Merck Millipore, Hertfordshire, England, UK). Slides were examined on an Olympus fluorescent microscope, and images were taken with a digital camera.

### 2.4 Extraction of protein from different cell compartments

To extract proteins in different cellular compartments to analyze the cellular location of the DRIM protein and other proteins, we employed a protein fractionation kit from Thermo Fisher (78840, Subcellular Protein Fractionation Kit for Cultured Cells) (Dublin, Republic Ireland). This allowed the separation of the cytoplasmic, membrane, nucleus soluble, chromatin‐bound, and the cytoskeletal fractions of proteins from the endothelial cells (El‐Daher et al., 2018; Garcia‐Del rio et al., 2023). The procedure was completed by strictly following instructions from the manufacturer.

### 2.5 Co‐immunoprecipitation (CO‐IP)

Total proteins at the same concentration was aliquoted into 1.5 mL microfuge tubes before adding the appropriate primary antibodies. The tubes were placed on a slow spinning wheel (25 rpm) at 4°C overnight before adding the agarose conjugated with Protein A/G (Santa‐Cruz Biotechnologies, SC‐2003). The mixtures were on the spinning wheel for a further 2 h and then placed in a microfuge to collect the protein‐antibody‐agarose mixture. For protein electrophoresis, the mixture was washed five times in the lysis buffer before added with 2‐ME containing sample buffer and boiling at 100°C. For proteomics analysis, proteins from the agarose bead mixture were first eluted from agarose using a glycine buffer (100 mM, pH 2.5–3.0) and, after removing the agarose, neutralized the protein to pH 7.2 with a Tris buffer.

### 2.6 Western blot analysis

Cells were harvested and lysed in a RIPA lysis buffer (50 mM Tris HCl, 150 mM NaCl, 1% NP‐40, 0.5% Sodium Deoxycholate, 1.0 mM EDTA, 0.1% SDS and 0.01% sodium azide, pH of 7.4). Protein concentrations were standardized to the same, then mixed with a 2‐meraptoethnol (2ME) containing sample loading buffer and boiled at 100°C. For fractionated proteins, the proteins were mixed with sample buffer and boiled. The proteins were loaded and separated using SDS‐PAGE. The proteins were then transferred to 0.45 μM PVDF membrane (Merck Millipore) for further analysis. After blocking with a skimmed milk mixture, the membrane was incubated overnight with the appropriate primary antibody, washed, and incubated with the secondary antibody conjugated with HRP. The proteins were visualized using the EZ‐ECL solution (Geneflow Ltd., Staffordshire, England, UK), and images were captured using a G‐BOX detection system (Syngene, Cambridge, England, UK).

### 2.7 Kinexus technology for proteomics

To determine the cellular protein response to DRIM. The present study used the platform service at Kinexus Bioinformatics (www.kinexus.ca.com). The KAM2023p protein array has more than 2000 antibodies being detected. Briefly, total proteins from endothelial cells, control, and cells with DRIM knockdown, were extracted and standardized to the same. Proteins were covalently labeled with a fluorescence dye. After removing the un‐bound free dye by gel filtration, the protein samples were loaded onto the antibody array and pre‐blocked for nonspecific binding. Following incubation and removal of unbound proteins, the array was scanned using a laser scanner to obtain the image of the respective protein samples. Bioinformatic analyses were conducted using the appropriate analysis tools provided by Kinexus Bioinformatics. For DRIM interactive proteins, the proteins eluted from the anti‐DRIM antibody immunoprecipitates were similarly applied to the Kinexus protein arrays.

### 2.8 Gene transcript analysis by polymerase chain reactions

Total RNA was extracted using the TRI Reagent (Sigma‐Aldrich). Reverse transcription of the RNA was then performed using a reverse transcription kit (Promega, Southampton, England, UK). Quantitative analysis of gene transcripts was conducted using a Step‐One Plus thermocycler from Fisher Scientific (Loughborough). Amplification chemistry employing a FAM tagged Uniprimer™ was used. The primers used in the study are listed in Table 1, and the reverse primer with a unique z‐sequence to complement Uniprimer™ is indicated in the table. β‐actin was used as a housekeeping control to normalize the data. Relative quantification of gene expression was performed using the 2^−ΔΔ^ CT method.

**Table 1:** List of q‐PCR primers.

	Forward (5′‐3′)	zReverse (5′‐3′)
DRIM	5′ACTGTCCTTCCTGTGATTGA3′	5′ACTGAACCTGACCGTACAAGAAAACTAAACAGAAGGCGA3′
Src	5′CTTCAACTCCTCGGACAC3′	5′ACTGAACCTGACCGTACATTTCTTGAAGGACAGGTCTG3′
β‐actin	5′CATTAAGGAGAAGCTGTGCT3′	5′ACTGAACCTGACCGTACAGCCATCCACAGTCTTCTG3′

*Note*: The underlined are the z‐sequence.

### 2.9 Vascular tubule formation assay

The Endothelial cells were cultured in DMEM medium containing 0.2% FBS for 24 h. Subconfluent cells were harvested and resuspended in medium (Medium 200 μL with 10% FCS). This suspension was seeded (80,000 cells/well) in growth factor‐reduced matrigel‐coated 24‐well plate (BD Bioscience) and incubated up to 8 h at 37°C to form capillary‐like tubule structures. Tubule formation was examined under an inverted microscope and photographed at 10× magnification. After incubation, the number of tubes and junctions of the tubular structures was quantified.

### 2.10 Cellular migration assay

Migration was evaluated using transwell migration method to quantify the ability of cells to migrate across 8 µm pore size membranes in 24‐well tissue culture plates (Millipore). Endothelial cells (2 × 10^4^) in 200 µL medium were added to the upper chamber of the transwell unit. The lower chamber contained 600 µL of complete culture medium with 10% FCS. The cells were incubated for 20 h at 37°C. The cells that had migrated through the membrane pores and stuck onto the surface of the lower chamber were first fixed with 4% formaldehyde, after removing the non‐migrated cells in the upper chamber. Migrated cells were then stained with crystal violet dye (0.5% w/v) before being quantified.

### 2.11 Electric cell‐substrate impedance sensing (ECIS) based cell migration assays

ECIS can be used as an alternative to conventional function assays including cell adhesion, cellular migration, and barrier function (Keese et al., 2004; Stolwijk et al., 2011). This was modified from a method previously described. Briefly, 96‐well microarrays (96W1E) were used with a ECIS instrument (Applied Biophysics Ltd, Troy, NY, USA). Endothelial cells were added to the wells of the 96W1E ECIS array and immediately placed on the ECIS instrument to track cell adhesion. This was done over a range of frequencies, namely from 1000 to 64,000 Hz. For cellular migration, confluent vascular endothelial cells monolayers in the arrays were first electrically wounded (2000 mA for 20 s each), after which the migration of the cells was immediately tracked, again over a range of frequencies. All the experiments were conducted in triplicate.

### 2.12 Statistical analysis

All statistical analyses were performed with the Statistical Package for Social Sciences for Windows version 17 software (SPSS). Data are shown as the mean ± standard deviation (SD) and two‐tailed unpaired Student's *t*‐tests were used to compare differences between two groups. Data that did not meet the criteria of normality test were analyzed by the Kruskal–Wallis test for more than two groups and Mann–Whitney's test for two groups. Paired comparisons were performed using Wilcoxon's test. A *p*‐value of <0.05 was considered statistically significant.

## 3 RESULTS

### 3.1 DRIM was abundantly expressed in vascular endothelial cells, and the DRIM protein was mainly located in nuclear and cytoskeletal fraction in vascular endothelial cells

**Figure 1:**
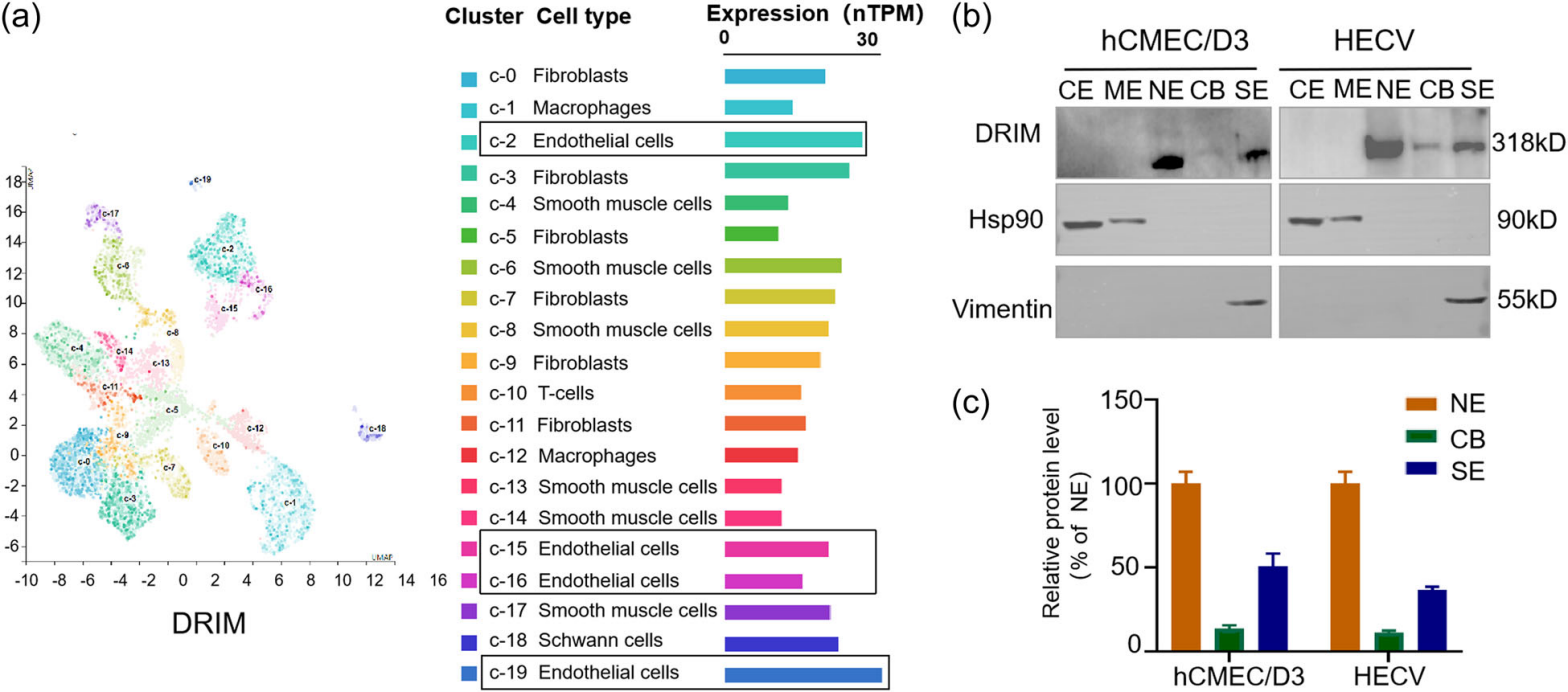
Intracellular distribution of DRIM proteins in vascular endothelial cells. (a) Vascular‐related single cell sequencing results. (b) After extracting proteins from different compartments of the cells, cells fractions were used for western blot analysis. HSP90 and vimentin were used as the respective positive controls for the cytosol and cytoskeletal proteins. (c) Relative quantification of DRIM in NE, CB, and SE using Image J. CB, chromatin‐bound fraction; CE, cytoplasm extraction; ME, membrane extraction; NE, nuclear soluble; SE, cytoskeletal fraction.

Vascular‐related single‐cell sequencing results suggest that DRIM has the highest expression value in vascular endothelial cells (https://www.proteinatlas.org/) (Figure 1a). To validate the cellular location of the DRIM protein in the vascular endothelial cells, a subcellular protein fractionation kit was chosen that allows extraction of proteins from different compartments of the cells, that is, the cytoplasmic fraction (CE), membrane fraction (ME), nuclear soluble fraction (NE), chromatin‐bound fraction (CB) and cytoskeletal protein fraction (SE). These proteins were subsequently applied to SDS‐PAGE gels. DRIM protein was clearly detected in nuclear soluble (NE), chromatin‐bound fraction (CB), and most notably, the cytoskeletal fraction (SE), from two different vascular endothelial cells cell lines (Figure 1b,c). Here, a known cytosolic protein HSP90 and a known cytoskeletal protein vimentin were respectively used a control for cytosolic and cytoskeletal protein for their cellular distributions. Vimentin was only found in the cytoskeletal fraction and HSP90 in the cytosolic and membrane fractions of the cellular fractions (Figure 1b). This finding thus shows that whilst the DRIM protein is a nuclear protein, as expected, it also strongly and constantly presents in the cytoskeletal fraction as seen in both cells. This would suggest that it has different and wider functions than initially anticipated.

### 3.2 Knockdown DRIM impacted biological functions and tubule formation of vascular endothelial cells

**Figure 2:**
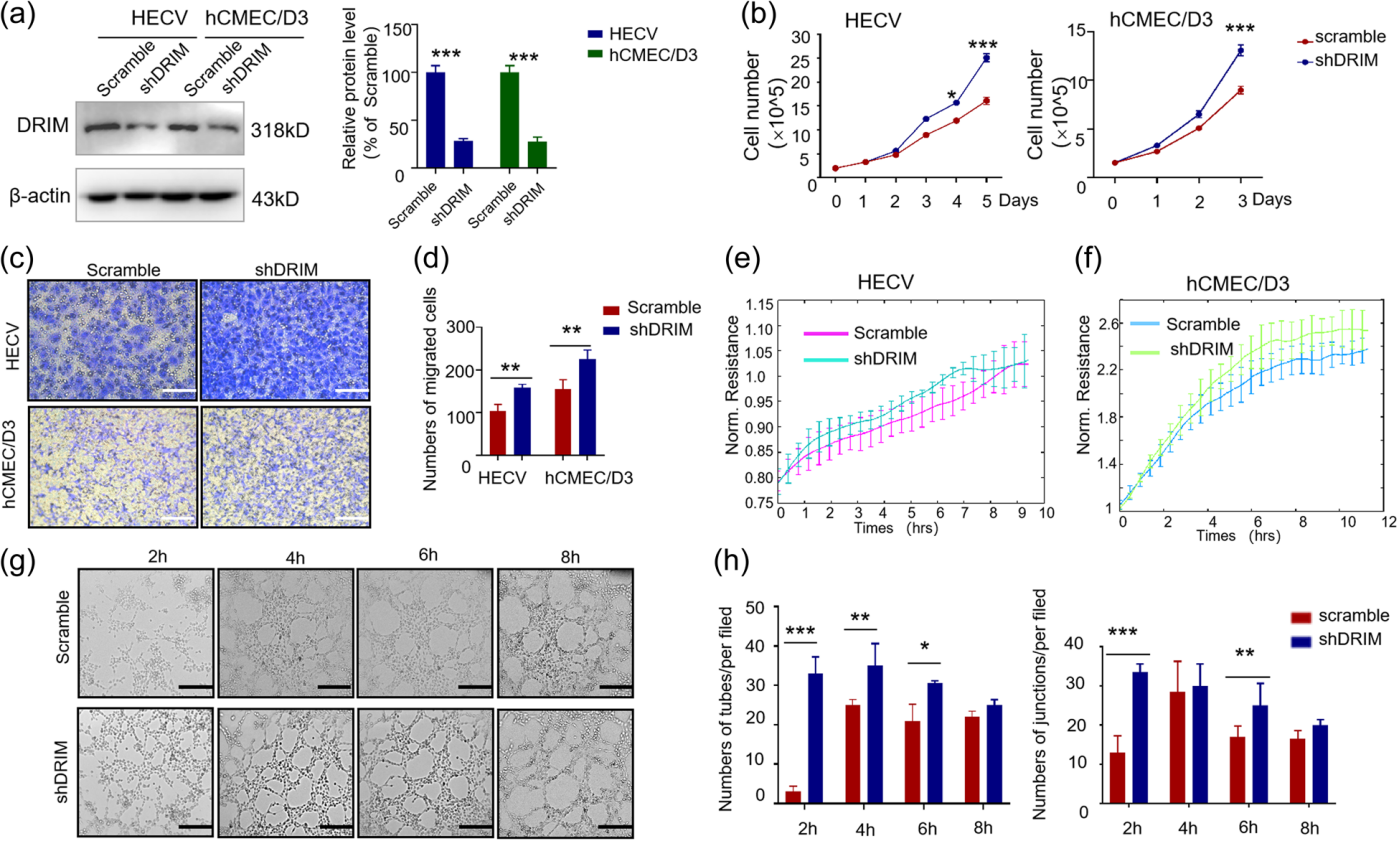
Knockdown DRIM promotes proliferation, migration, and microtubule formation of vascular endothelial cells. HECV and hCMEC/D3 cells were infected with indicated lentivirus expressing shRNA. After 72 h, HECV and hCMEC/D3 cells were used for western blot analysis, and relative quantification of DRIM protein was performed using Image J (a). Functional experiments were performed after knocking down DRIM in HECV and hCMEC/D3 cells, including cell counting (b), transwell assay (c, d), ECIS migration assay (e, f), microtubule formation assay (g, h). Data are shown as mean ± SD (*n* = 3). *p < .05; **p < .01; ***p < .001. Scale bars 100 μm in HECV (c), scale bars 200 μm in hCMEC/D3 (c), scale bars 200 μm in HECV and hCMEC/D3 (g).

To investigate the role of endogenous DRIM in vitro, lentiviral anti‐DRIM shRNA was used to create DRIM knockdown endothelial cell models (Figure 2a). Cell function assays including proliferation, migration and in vitro angiogenesis (vascular tubule formation) were assessed. HECV and hCMEC/D3 cells showed significant increase in their growth following DRIM knockdown (Figure 2b). We used different methods to detect cell migration, including the Boyden chamber assay (Figure 2c,d) and ECIS based assay (Figure 2e,f). It was found that cell migration significantly increased after knocking down of DRIM. Silencing of DRIM enhanced the ability of HECVs to form angiogenic tubules and junctions when compared with control cells (Figure 2g,h).

### 3.3 Hub proteins are associated with protein phosphorylation

**Figure 3:**
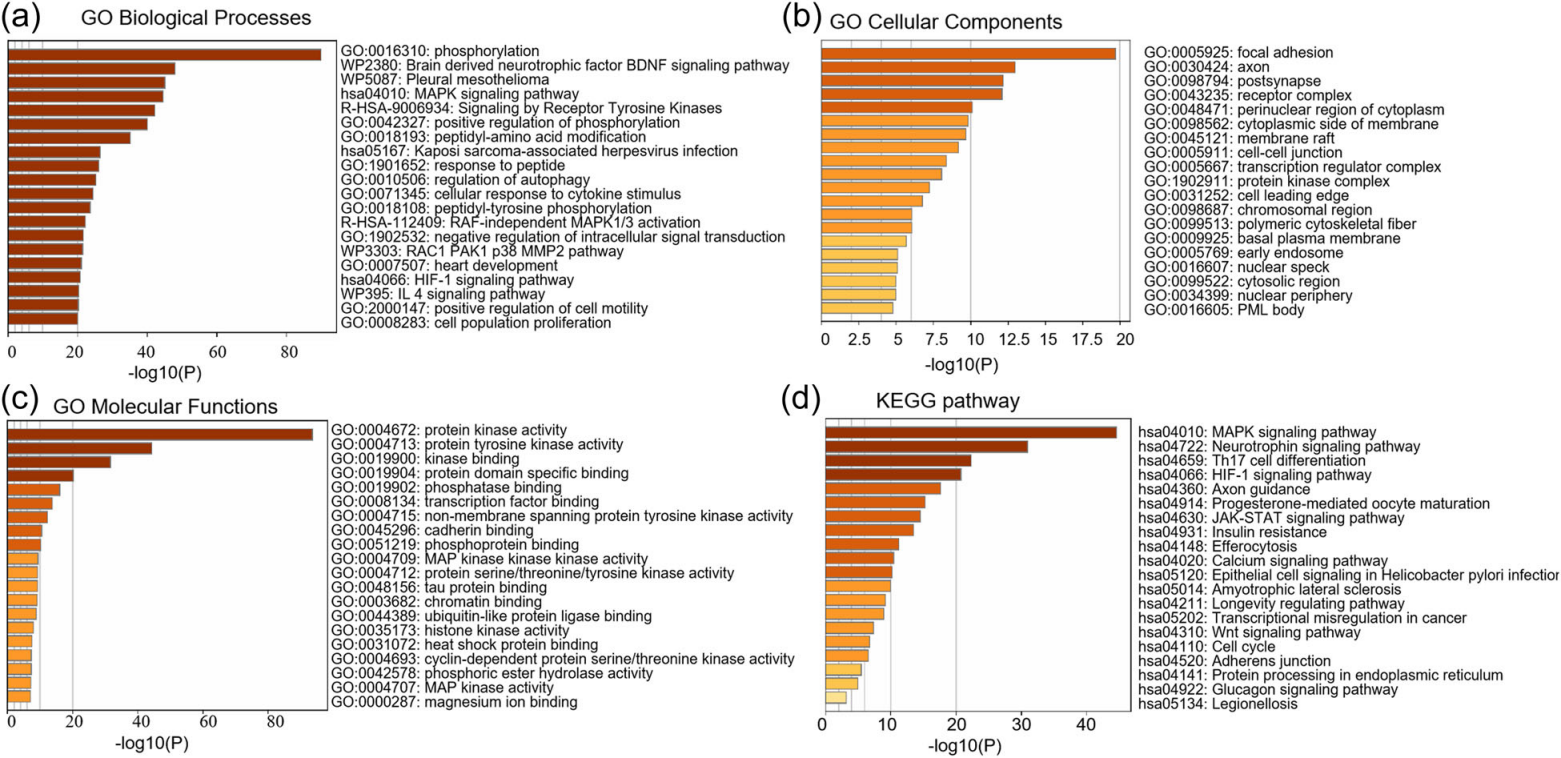
Functional analyses of the hub genes in response to DRIM knockdown. (a) Enriched biological processes among the hub genes. (b) Enriched cellular components among the hub genes. (c) Enriched molecular functions among the hub genes. (d) Enriched KEGG pathways among the hub genes.

To determine which protein changes occurred in response to DRIM knockdown, we utilized the Kinexus™ proteomic platform (Vancouver, British Columbia, Canada). We selected the TOP 200 upregulated proteins (Supporting Information S1: Table 1) after silencing DRIM in HECV cells for enrichment analysis (https://metascape.org/gp/index.html#/main/step1). GO and KEGG pathway enrichment analyses of the hub revealed a significant enrichment of protein phosphorylation (Figure 3a–c). Knocking down DRIM affected the MAPK pathway, JAK/STAT3 pathway, and focal adhesion, which is related with cell growth, migration, and angiogenesis (Figure 3d).

### 3.4 The direct interaction between Src and DRIM in vascular endothelial cells

**Figure 4:**
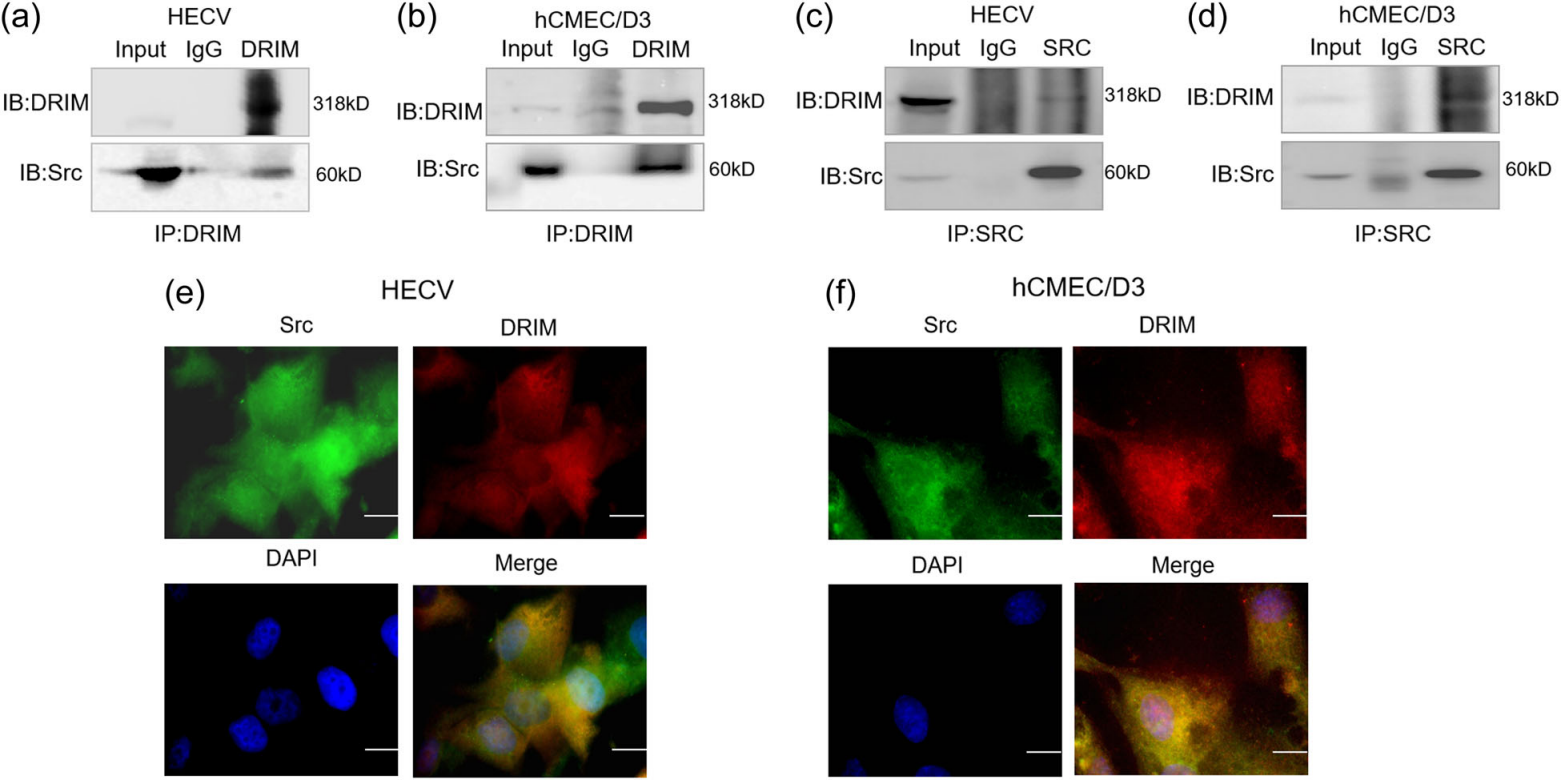
Src has direct interaction with DRIM in vascular endothelial cells. (a, b) Whole‐cell lysates (WCL) of HECV and hCMEC/D3 cells were subjected to Co‐IP with IgG (control) and anti‐DRIM antibodies. Western blot analysis for DRIM and Src in vascular endothelial cells. (c, d) Whole‐cell lysates (WCL) of HECV and hCMEC/D3 cells were subjected to Co‐IP with IgG (control) and anti‐Src antibodies. Western blot analysis for DRIM and Src in vascular endothelial cells. (e, f) Immunofluorescence staining of DRIM and Src in vascular endothelial cells. Scale bars 10 μm.

To explore the mechanisms underlying DRIM action, we used DRIM antibodies for co‐immunoprecipitation to enrich proteins for identification of potential binding partners of DRIM in HECV cells, followed by proteomics analysis to identify the partners for DRIM in HECV cells. Src protein was one of the most significantly enriched proteins in the proteomics assays. Src protein serves as a link between membrane receptors and cytoplasmic signaling mechanisms to regulate various basic cellular processes. It is generally understood that the activation of the tyrosine kinase Src (Brunton & Frame, 2008; Seong et al., 2011; Avizienyte & Frame, 2005) and its downstream signaling molecules (Heerkens et al., 2007; Miki et al., 2000; Schneider et al., 2008) are required for the polymerization of branched actin meshwork and the initiation of membrane protrusion. GO and KEGG pathway enrichment analysis also indicated that Src kinase may be involved in the changed processes and pathways. We identified Src as a DRIM binding partner, which was verified by co‐IP (Figure 4a–d) and immunofluorescence staining of DRIM and Src in vascular endothelial cells. DRIM staining was seen in both nucleus and in the cytoplasmic region. DRIM and Src colocalized in both the nucleus and cytoplasm (Figure 4e,f).

### 3.5 Activation of the Src pathway following DRIM knockdown in vascular endothelial cells

**Figure 5:**
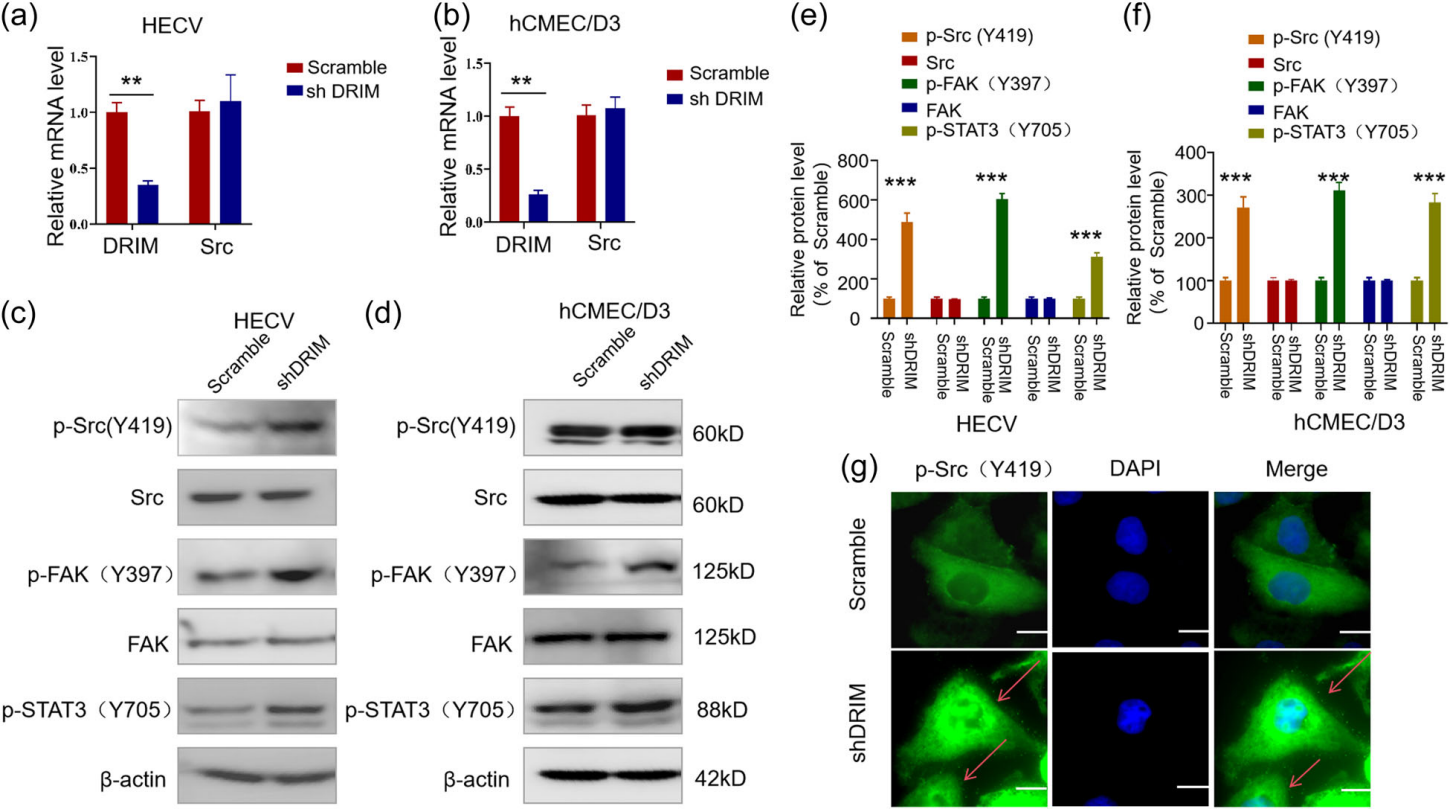
Silencing DRIM activates Src kinase pathway in vascular endothelial cells. HECV and hCMEC/D3 cells were infected with the DRIM shRNAs. After 72 h, cells were harvested for q‐PCR (a,b) and western blot analysis (c, d). (e, f) Relative quantification of proteins in (c, d) using Image J. (g) Immunofluorescence staining of p‐Src (Y419) in HECV cells. The red arrows point to the activated Src in cell membrane. **p < .01, ***p < .001. Scale bars 10 μm.

Src contains multiple structural domains. The catalytic domain SH1 contains atyrosine phosphorylation site Tyr419, which causes Src to auto‐phosphorylate. DRIM knockdown did not affect Src mRNA levels and protein levels in vascular endothelial cell lines (Figure 5a–d). Notably, the Src kinase pathway was activated following DRIM silencing, as seen by a significant increase in levels of phosphorylated Src (Y419). Activation of Src and the Src pathway are known to activate the focal adhesion kinase (FAK) signal pathway. This was indeed seen in the present study in that DRIM silencing and the subsequent activation of Src lead to activation of the FAK phosphorylation (Y397) and STAT3 (Y705) (Figure 5c–f). Src is activated at the cell membrane, the majority of activated Src molecules are localized at focal adhesions (Machiyama et al., 2017). This change was also visualized using immunofluorescence staining of phosphorylated Src (Y419) in HECV cells with the anti‐phospho‐Src (Y419) antibody. As shown in Figure 5g, phosphorylated Src (Y419) was indeed significantly increased in the cell membrane after knocking down DRIM in HECV cells (Figure 5g).

### 3.6 Suppression of Src reverses cellular phenotypic changes caused by silencing DRIM in vascular endothelial cells

**Figure 6:**
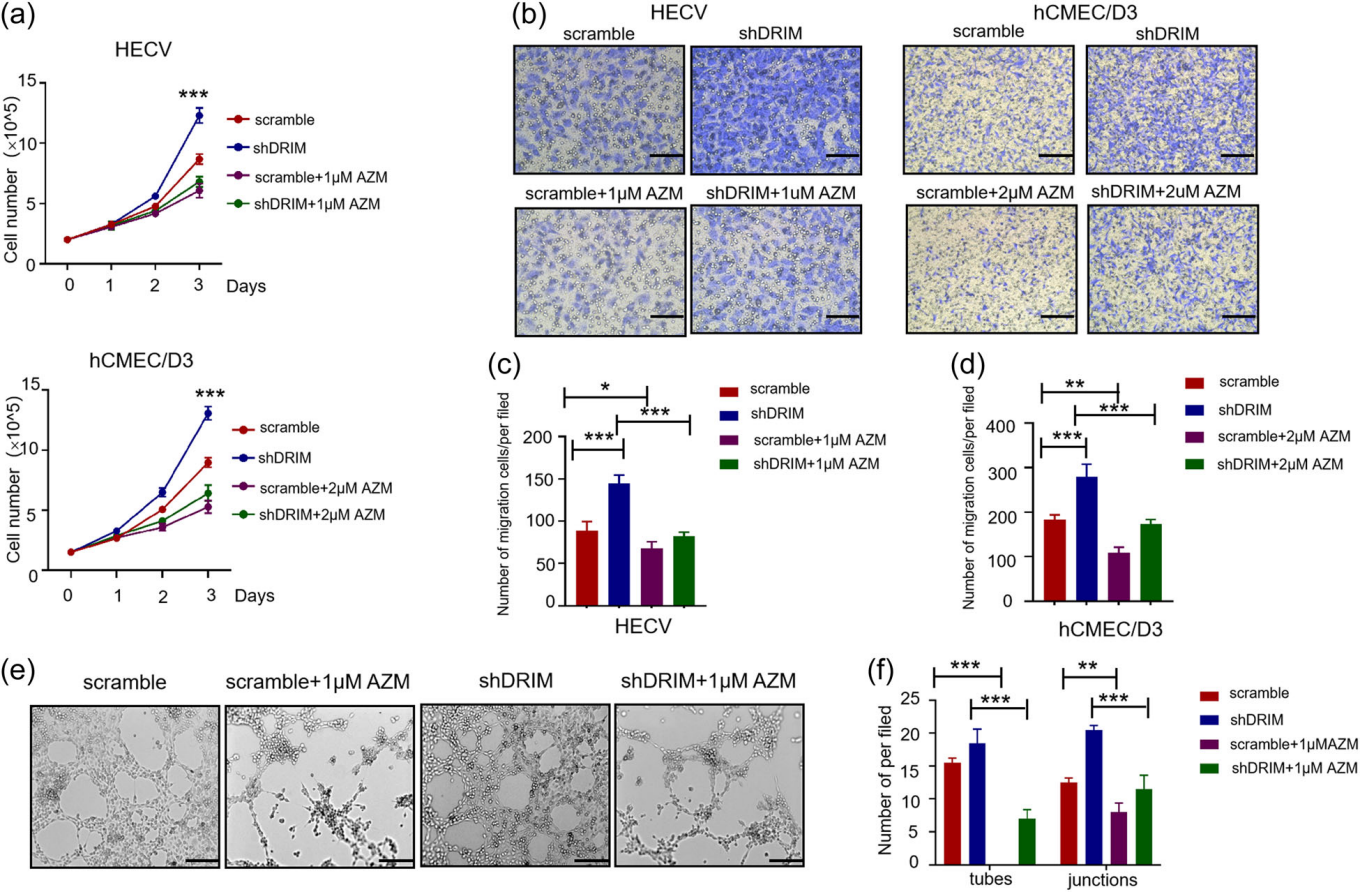
Src inhibition and the cellular phenotype caused by silencing DRIM in vascular endothelial cells. HECV and hCMEC/D3 cells were infected with the indicated lentivirus. After 72 h, vascular endothelial cells with DRIM‐knockdown were treated with AZM475271 continuously and then used for cell growth assay (a, b). The DRIM‐knockdown and scramble vascular endothelial cells were treated with AZM475271 for 4 h and then used for the transwell migration assay (b–d), and microtubule formation assay (e, f). Data are shown as mean ± SD (*n* = 3). *p < .05; **p < .01; ***p < .001. Scale bars 100 μm in HECV(b), scale bars 200 μm in hCMEC/D3 (b), scale bars 200 μm in HECV and hCMEC/D3 (e).

To verify whether the changes in cell phenotype after knocking down of DRIM are directly related to the activation of the Src kinase pathway, we used AZM475271, a potent and selective Src kinase inhibitor, to treat the vascular endothelial cells and then observed changes in cell phenotypes. AZM475271 reversed cellular phenotypic changes that were caused by silencing DRIM in HECV and hCMEC/D3, including cell growth (Figure 6a), cell migration (Figure 6b–d), and tubule formation (Figure 6e,f). When HECV^shDRIM^ and hCMEC/D3^shDRIM^ cells were treated with AZD475271, the growth and the migration of cells were significantly decreased. Figure 6a showed the significant difference (*p* < .001) of cell growth between the shDRIM group and the shDRIM+AZM475271 group in HECV and hCMEC/D3 cells. There was also a significant difference between the scramble group and the scramble+AZM475271 group (*p* < .001). After continuous treatment of AZM475271 in vascular endothelial cells for 3 days, there was no significant difference in cell growth between the vascular endothelial cells with or without DRIM knockdown, which indicated the inhibition of the Src signaling pathway can reverse the cell growth phenotypic changes caused by knockdown of DRIM. In the transwell migration assay (Figure 6b–d), it showed a similar tendency with the cell growth. HECV cells were pretreated with AZM475271 for 4 h and then subjected to vascular tubule formation assay. Significantly, AZM475271 treatment in HECV cells directly inhibited tubule formation in both HECV^scramble^ and HECV^shDRIM^ (Figure 6e,f). These results further indicated that the pivotal role of Src signaling pathway in vascular endothelial cells and DRIM modulated the activation of Src signaling in vascular endothelial cells.

## 4 DISCUSSION

DRIM was first described as being downregulated in metastatic breast cancer cells. Previous results suggest that DRIM is a nucleolar protein and that it activates RNA polymerase I transcription and thereby affects the acetylation of upstream binding factor (Learned et al., 1985; Learned et al., 1986). Despite its initial description two decades ago, information regarding DRIM's role in vascular endothelial cells remains scarce. In this study, we explored the localization of the DRIM protein‐an entity closely linked to cancer progression and found it to be predominantly present in the nuclear soluble fraction, chromatin‐bound fraction, and notably within the cytoskeletal fraction of vascular endothelial cells. This presence directly influenced Src kinase activation and modulated processes such as endothelial cell proliferation, migration, and angiogenesis.

While traditionally regarded primarily as a nuclear protein, our findings revealed that DRIM was observable not only in the nucleus (mainly within nuclear soluble fractions and weakly associated with chromatin) but also significantly within the cytoskeletal compartment. Immunofluorescence studies corroborated this distribution pattern by showing DRIM staining both in cytoplasmic regions and nucleus. Thus, we concluded that DRIM served dual roles as both a nuclear and cytoskeletal‐associated protein in endothelial cells, implying distinct functional capacities across various cellular compartments.

In terms of its interaction network with other proteins, knowledge about potential interacting partners for DRIM is limited. Previous indications suggest that PPP1R26 (KIAA0649) may interact with DRIM (Xing et al., 2005). To investigate further potential partner proteins interacting with DRIM within both cytoplasmic and nuclear fractions of these cells, an immunoprecipitation study was conducted (data not shown). Proteins precipitated by DRIM were analyzed using proteomics platforms, revealing several proteins with diverse functions associated with it, which suggests collaborative roles for DRIM alongside key partners involved in vascular endothelial cell biology.

Src family kinases are known to regulate numerous cellular activities including proliferation and oncogenesis. Both C‐Src and its retroviral counterpart V‐Src are classified as non‐receptor tyrosine kinases belonging to protein tyrosine kinase families (PTK). Under normal physiological conditions, Src maintains an inactive conformation while anchored on the inner surface of the plasma membrane through acylation modifications at its amino terminus, here it transmits signals between surface receptors and intracellular signaling molecules or transcription factors. The autonomous phosphorylation at tyrosine 419 represents a primary mechanism for activating Src proteins. As for DRIM, there is currently a limited amount of acknowledgment and research regarding its implications in cancer and other diseases. However, in this study, we have identified Src as a binding partner for DRIM, specifically noting that reduced levels of DRIM led to increased phosphorylation at tyrosine 419, which subsequently activates SRC kinase pathways.

Cytoskeletal reorganization along with cell migration constitutes fundamental events during angiogenesis‐a process relevant not only to wound healing but also implicated in pathological states like cancer or cardiovascular diseases (Arora et al., 2022). Numerous investigations highlight Src's critical involvement in angiogenesis, suggesting it could serve as a viable target for antiangiogenic therapies (Liang et al., 2010; Liu et al., 2022). Given its proposed essential function during what is termed “angiogenic switch,” Src likely influences angiogenesis through multiple mechanisms including regulation over gene expression related to growth factors such as VEGF or cytokines like IL‐8 (Kanda et al., 2007; Mukhopadhyay et al., 1995; Summy et al., 2005; Zhang et al., 2013). Notably, FAK activation plays an integral role in facilitating protrusion formation among endothelial cells where polarized FAK distribution critically determines directional migration patterns (Hu et al., 2014). Furthermore, Src can influence cytoskeleton rearrangement via phosphorylating FAK, thereby regulating adhesion properties necessary for effective migration. It is plausible to suggest that loss of DRIM would result in Src activation (phosphorylation) and promotion of the SRC/FAK complex in the regulation of the migration and angiogenesis of vascular endothelial cells.

In the present study, we have shown that knockdown of DRIM is associated with increased angiogenic formation and migration capacity of endothelial cells in vitro. Future studies employing a vascular endothelial cells‐specific DRIM‐knockout mouse model would be required to pinpoint in detail the molecular functions of DRIM in endothelial cells in vivo. It will be intriguing to study the role of DRIM in tumor angiogenesis and explore its therapeutic potential against tumor growth and metastasis.

**Figure 7:**
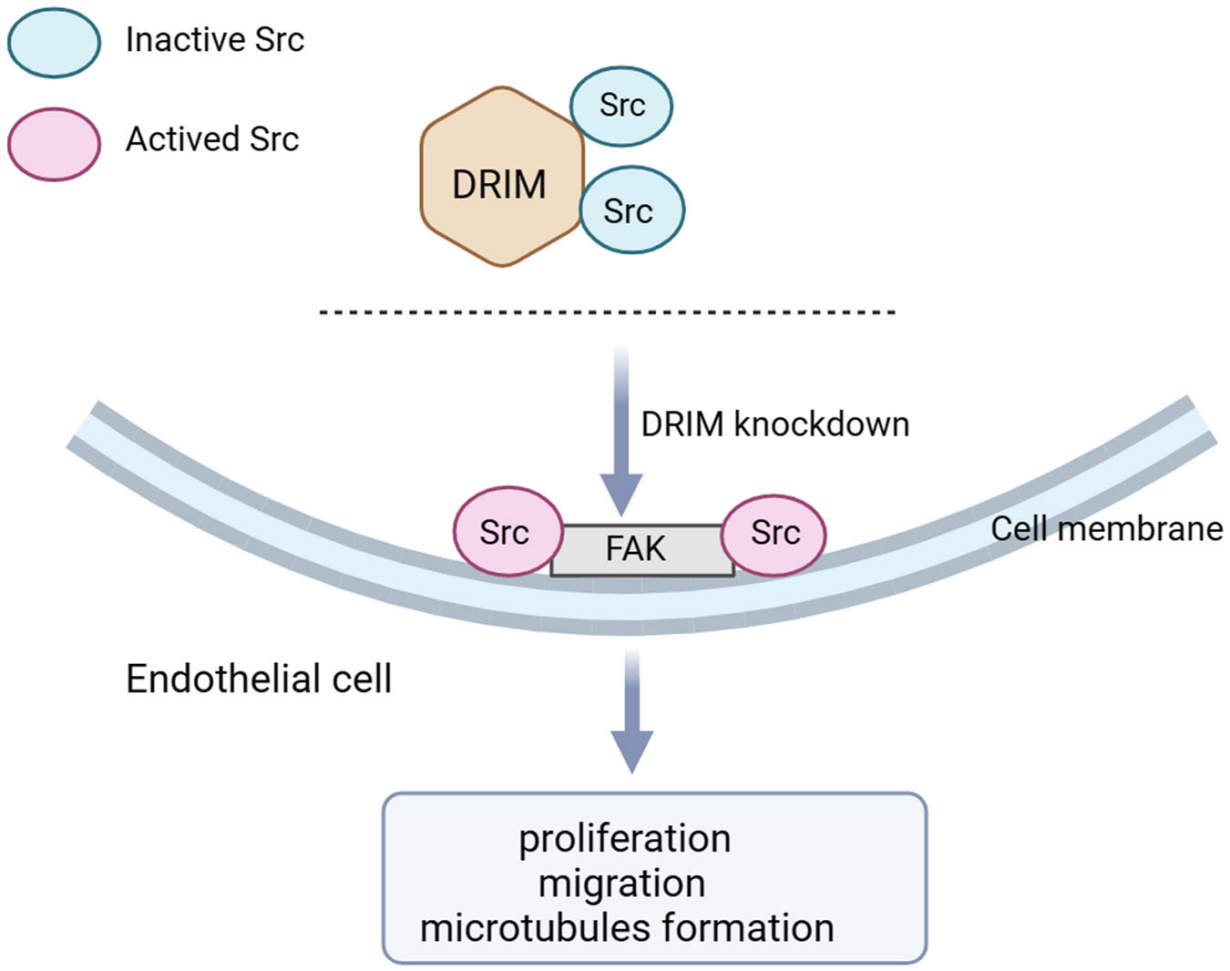
Proposed mechanism of DRIM in endothelial cells. Silencing DRIM can activate the tyrosine 419 site phosphorylation of Src kinase in endothelial cells, thereby affecting the downstream proteins of Src including p‐FAK and other proteins, and promoting cell growth, migration, and microtubule formation.

To conclude, this study is the first to report the role of DRIM in endothelial cell function and angiogenesis. These results are summarized in Figure 7. Our findings suggest that DRIM executes important functions in endothelial cells, with significant impacts on Src signaling, cell proliferation, migration, and angiogenic capacity.

## AUTHOR CONTRIBUTIONS

Wenguo Jiang and Bo Dong conceived, designed, and directed the studies. Jia Tong, Xuefeu Dong, and Yiming Ynag performed q‐PCR, Western blot analysis, and function experiments. Jia Tong and Tracey A Martin performed the statistical analysis. Jia Tong performed co‐IP and immunofluorescence and wrote the manuscript with assistance from all the authors.

## CONFLICT OF INTEREST STATEMENT

The authors declare no conflicts of interest.

## Supporting information

Supporting information.

## Data Availability

The data that support the findings of this study are available from the corresponding author upon reasonable request.
