# Supplementary material for: DRIM modulates Src activation and regulates angiogenic functions in vascular endothelial cells
Source: Cell Biol Int. 2024 Dec 8;49(3):277–87. doi: 10.1002/cbin.12265 (PMC11811745; doi:10.1002/cbin.12265)
Supplement: Supplementary file 1 — Supporting information. [file CBIN-49-277-s001.docx]

| \| supplementary TABLE 1 \| \| \| \| --- \| --- \| --- \| \| Uniprot ID \| Entry Name \| Protein names \| \| O00203 \| AP3B1_HUMAN \| AP-3 complex subunit beta-1 (Adaptor protein complex AP-3 subunit beta-1) (Adaptor-related protein complex 3 subunit beta-1) (Beta-3A-adaptin) (Clathrin assembly protein complex 3 beta-1 large chain) \| \| O00273 \| DFFA_HUMAN \| DNA fragmentation factor subunit alpha (DNA fragmentation factor 45 kDa subunit) (DFF-45) (Inhibitor of CAD) (ICAD) \| \| O00506 \| STK25_HUMAN \| Serine/threonine-protein kinase 25 (EC 2.7.11.1) (Ste20-like kinase) (Sterile 20/oxidant stress-response kinase 1) (SOK-1) (Ste20/oxidant stress response kinase 1) \| \| O14974 \| MYPT1_HUMAN \| Protein phosphatase 1 regulatory subunit 12A (Myosin phosphatase-targeting subunit 1) (Myosin phosphatase target subunit 1) (Protein phosphatase myosin-binding subunit) \| \| O15357 \| SHIP2_HUMAN \| Phosphatidylinositol 3,4,5-trisphosphate 5-phosphatase 2 (EC 3.1.3.86) (Inositol polyphosphate phosphatase-like protein 1) (INPPL-1) (Protein 51C) (SH2 domain-containing inositol 5'-phosphatase 2) (SH2 domain-containing inositol phosphatase 2) (SHIP-2) \| \| O15530 \| PDPK1_HUMAN \| 3-phosphoinositide-dependent protein kinase 1 (hPDK1) (EC 2.7.11.1) \| \| O43251 \| RFOX2_HUMAN \| RNA binding protein fox-1 homolog 2 (Fox-1 homolog B) (Hexaribonucleotide-binding protein 2) (RNA-binding motif protein 9) (RNA-binding protein 9) (Repressor of tamoxifen transcriptional activity) \| \| O43353 \| RIPK2_HUMAN \| Receptor-interacting serine/threonine-protein kinase 2 (EC 2.7.11.1) (CARD-containing interleukin-1 beta-converting enzyme-associated kinase) (CARD-containing IL-1 beta ICE-kinase) (RIP-like-interacting CLARP kinase) (Receptor-interacting protein 2) (RIP-2) (Tyrosine-protein kinase RIPK2) (EC 2.7.10.2) \| \| O43426 \| SYNJ1_HUMAN \| Synaptojanin-1 (EC 3.1.3.36) (Synaptic inositol 1,4,5-trisphosphate 5-phosphatase 1) \| \| O43524 \| FOXO3_HUMAN \| Forkhead box protein O3 (AF6q21 protein) (Forkhead in rhabdomyosarcoma-like 1) \| \| O60674 \| JAK2_HUMAN \| Tyrosine-protein kinase JAK2 (EC 2.7.10.2) (Janus kinase 2) (JAK-2) \| \| O75096 \| LRP4_HUMAN \| Low-density lipoprotein receptor-related protein 4 (LRP-4) (Multiple epidermal growth factor-like domains 7) \| \| O75116 \| ROCK2_HUMAN \| Rho-associated protein kinase 2 (EC 2.7.11.1) (Rho kinase 2) (Rho-associated, coiled-coil-containing protein kinase 2) (Rho-associated, coiled-coil-containing protein kinase II) (ROCK-II) (p164 ROCK-2) \| \| O75582 \| KS6A5_HUMAN \| Ribosomal protein S6 kinase alpha-5 (S6K-alpha-5) (EC 2.7.11.1) (90 kDa ribosomal protein S6 kinase 5) (Nuclear mitogen- and stress-activated protein kinase 1) (RSK-like protein kinase) (RSKL) \| \| O94804 \| STK10_HUMAN \| Serine/threonine-protein kinase 10 (EC 2.7.11.1) (Lymphocyte-oriented kinase) \| \| O95292 \| VAPB_HUMAN \| Vesicle-associated membrane protein-associated protein B/C (VAMP-B/VAMP-C) (VAMP-associated protein B/C) (VAP-B/VAP-C) \| \| O95819 \| M4K4_HUMAN \| Mitogen-activated protein kinase kinase kinase kinase 4 (EC 2.7.11.1) (HPK/GCK-like kinase HGK) (MAPK/ERK kinase kinase kinase 4) (MEK kinase kinase 4) (MEKKK 4) (Nck-interacting kinase) \| \| O96013 \| PAK4_HUMAN \| Serine/threonine-protein kinase PAK 4 (EC 2.7.11.1) (p21-activated kinase 4) (PAK-4) \| \| P00439 \| PH4H_HUMAN \| Phenylalanine-4-hydroxylase (PAH) (EC 1.14.16.1) (Phe-4-monooxygenase) \| \| P00533 \| EGFR_HUMAN \| Epidermal growth factor receptor (EC 2.7.10.1) (Proto-oncogene c-ErbB-1) (Receptor tyrosine-protein kinase erbB-1) \| \| P01106 \| MYC_HUMAN \| Myc proto-oncogene protein (Class E basic helix-loop-helix protein 39) (bHLHe39) (Proto-oncogene c-Myc) (Transcription factor p64) \| \| P01112 \| RASH_HUMAN \| GTPase HRas (EC 3.6.5.2) (H-Ras-1) (Ha-Ras) (Transforming protein p21) (c-H-ras) (p21ras) [Cleaved into: GTPase HRas, N-terminally processed] \| \| P02545 \| LMNA_HUMAN \| Prelamin-A/C [Cleaved into: Lamin-A/C (70 kDa lamin) (Renal carcinoma antigen NY-REN-32)] \| \| P04035 \| HMDH_HUMAN \| 3-hydroxy-3-methylglutaryl-coenzyme A reductase (HMG-CoA reductase) (EC 1.1.1.34) \| \| P04049 \| RAF1_HUMAN \| RAF proto-oncogene serine/threonine-protein kinase (EC 2.7.11.1) (Proto-oncogene c-RAF) (cRaf) (Raf-1) \| \| P04083 \| ANXA1_HUMAN \| Annexin A1 (Annexin I) (Annexin-1) (Calpactin II) (Calpactin-2) (Chromobindin-9) (Lipocortin I) (Phospholipase A2 inhibitory protein) (p35) [Cleaved into: Annexin Ac2-26] \| \| P04626 \| ERBB2_HUMAN \| Receptor tyrosine-protein kinase erbB-2 (EC 2.7.10.1) (Metastatic lymph node gene 19 protein) (MLN 19) (Proto-oncogene Neu) (Proto-oncogene c-ErbB-2) (Tyrosine kinase-type cell surface receptor HER2) (p185erbB2) (CD antigen CD340) \| \| P04637 \| P53_HUMAN \| Cellular tumor antigen p53 (Antigen NY-CO-13) (Phosphoprotein p53) (Tumor suppressor p53) \| \| P04792 \| HSPB1_HUMAN \| Heat shock protein beta-1 (HspB1) (28 kDa heat shock protein) (Estrogen-regulated 24 kDa protein) (Heat shock 27 kDa protein) (HSP 27) (Stress-responsive protein 27) (SRP27) \| \| P05067 \| A4_HUMAN \| Amyloid-beta precursor protein (APP) (ABPP) (APPI) (Alzheimer disease amyloid A4 protein homolog) (Alzheimer disease amyloid protein) (Amyloid precursor protein) (Amyloid-beta (A4) precursor protein) (Amyloid-beta A4 protein) (Cerebral vascular amyloid peptide) (CVAP) (PreA4) (Protease nexin-II) (PN-II) [Cleaved into: N-APP; Soluble APP-alpha (S-APP-alpha); Soluble APP-beta (S-APP-beta); C99 (Beta-secretase C-terminal fragment) (Beta-CTF); Amyloid-beta protein 42 (Abeta42) (Beta-APP42); Amyloid-beta protein 40 (Abeta40) (Beta-APP40); C83 (Alpha-secretase C-terminal fragment) (Alpha-CTF); P3(42); P3(40); C80; Gamma-secretase C-terminal fragment 59 (Amyloid intracellular domain 59) (AICD-59) (AID(59)) (Gamma-CTF(59)); Gamma-secretase C-terminal fragment 57 (Amyloid intracellular domain 57) (AICD-57) (AID(57)) (Gamma-CTF(57)); Gamma-secretase C-terminal fragment 50 (Amyloid intracellular domain 50) (AICD-50) (AID(50)) (Gamma-CTF(50)); C31] \| \| P06493 \| CDK1_HUMAN \| Cyclin-dependent kinase 1 (CDK1) (EC 2.7.11.22) (EC 2.7.11.23) (Cell division control protein 2 homolog) (Cell division protein kinase 1) (p34 protein kinase) \| \| P07332 \| FES_HUMAN \| Tyrosine-protein kinase Fes/Fps (EC 2.7.10.2) (Feline sarcoma/Fujinami avian sarcoma oncogene homolog) (Proto-oncogene c-Fes) (Proto-oncogene c-Fps) (p93c-fes) \| \| P08238 \| HS90B_HUMAN \| Heat shock protein HSP 90-beta (HSP 90) (Heat shock 84 kDa) (HSP 84) (HSP84) \| \| P08581 \| MET_HUMAN \| Hepatocyte growth factor receptor (HGF receptor) (EC 2.7.10.1) (HGF/SF receptor) (Proto-oncogene c-Met) (Scatter factor receptor) (SF receptor) (Tyrosine-protein kinase Met) \| \| P09104 \| ENOG_HUMAN \| Gamma-enolase (EC 4.2.1.11) (2-phospho-D-glycerate hydro-lyase) (Enolase 2) (Neural enolase) (Neuron-specific enolase) (NSE) \| \| P09429 \| HMGB1_HUMAN \| High mobility group protein B1 (High mobility group protein 1) (HMG-1) \| \| P09619 \| PGFRB_HUMAN \| Platelet-derived growth factor receptor beta (PDGF-R-beta) (PDGFR-beta) (EC 2.7.10.1) (Beta platelet-derived growth factor receptor) (Beta-type platelet-derived growth factor receptor) (CD140 antigen-like family member B) (Platelet-derived growth factor receptor 1) (PDGFR-1) (CD antigen CD140b) \| \| P09651 \| ROA1_HUMAN \| Heterogeneous nuclear ribonucleoprotein A1 (hnRNP A1) (Helix-destabilizing protein) (Single-strand RNA-binding protein) (hnRNP core protein A1) [Cleaved into: Heterogeneous nuclear ribonucleoprotein A1, N-terminally processed] \| \| P10636 \| TAU_HUMAN \| Microtubule-associated protein tau (Neurofibrillary tangle protein) (Paired helical filament-tau) (PHF-tau) \| \| P10721 \| KIT_HUMAN \| Mast/stem cell growth factor receptor Kit (SCFR) (EC 2.7.10.1) (Piebald trait protein) (PBT) (Proto-oncogene c-Kit) (Tyrosine-protein kinase Kit) (p145 c-kit) (v-kit Hardy-Zuckerman 4 feline sarcoma viral oncogene homolog) (CD antigen CD117) \| \| P11021 \| BIP_HUMAN \| Endoplasmic reticulum chaperone BiP (EC 3.6.4.10) (78 kDa glucose-regulated protein) (GRP-78) (Binding-immunoglobulin protein) (BiP) (Heat shock protein 70 family protein 5) (HSP70 family protein 5) (Heat shock protein family A member 5) (Immunoglobulin heavy chain-binding protein) \| \| P11137 \| MTAP2_HUMAN \| Microtubule-associated protein 2 (MAP-2) \| \| P11142 \| HSP7C_HUMAN \| Heat shock cognate 71 kDa protein (EC 3.6.4.10) (Heat shock 70 kDa protein 8) (Lipopolysaccharide-associated protein 1) (LAP-1) (LPS-associated protein 1) \| \| P11168 \| GTR2_HUMAN \| Solute carrier family 2, facilitated glucose transporter member 2 (Glucose transporter type 2, liver) (GLUT-2) \| \| P11229 \| ACM1_HUMAN \| Muscarinic acetylcholine receptor M1 \| \| P12036 \| NFH_HUMAN \| Neurofilament heavy polypeptide (NF-H) (200 kDa neurofilament protein) (Neurofilament triplet H protein) \| \| P16104 \| H2AX_HUMAN \| Histone H2AX (H2a/x) (Histone H2A.X) \| \| P16234 \| PGFRA_HUMAN \| Platelet-derived growth factor receptor alpha (PDGF-R-alpha) (PDGFR-alpha) (EC 2.7.10.1) (Alpha platelet-derived growth factor receptor) (Alpha-type platelet-derived growth factor receptor) (CD140 antigen-like family member A) (CD140a antigen) (Platelet-derived growth factor alpha receptor) (Platelet-derived growth factor receptor 2) (PDGFR-2) (CD antigen CD140a) \| \| P16885 \| PLCG2_HUMAN \| 1-phosphatidylinositol 4,5-bisphosphate phosphodiesterase gamma-2 (EC 3.1.4.11) (Phosphoinositide phospholipase C-gamma-2) (Phospholipase C-IV) (PLC-IV) (Phospholipase C-gamma-2) (PLC-gamma-2) \| \| P17096 \| HMGA1_HUMAN \| High mobility group protein HMG-I/HMG-Y (HMG-I(Y)) (High mobility group AT-hook protein 1) (High mobility group protein A1) (High mobility group protein R) \| \| P17706 \| PTN2_HUMAN \| Tyrosine-protein phosphatase non-receptor type 2 (EC 3.1.3.48) (T-cell protein-tyrosine phosphatase) (TCPTP) \| \| P17752 \| TPH1_HUMAN \| Tryptophan 5-hydroxylase 1 (EC 1.14.16.4) (Tryptophan 5-monooxygenase 1) \| \| P17858 \| PFKAL_HUMAN \| ATP-dependent 6-phosphofructokinase, liver type (ATP-PFK) (PFK-L) (EC 2.7.1.11) (6-phosphofructokinase type B) (Phosphofructo-1-kinase isozyme B) (PFK-B) (Phosphohexokinase) \| \| P17948 \| VGFR1_HUMAN \| Vascular endothelial growth factor receptor 1 (VEGFR-1) (EC 2.7.10.1) (Fms-like tyrosine kinase 1) (FLT-1) (Tyrosine-protein kinase FRT) (Tyrosine-protein kinase receptor FLT) (FLT) (Vascular permeability factor receptor) \| \| P19793 \| RXRA_HUMAN \| Retinoic acid receptor RXR-alpha (Nuclear receptor subfamily 2 group B member 1) (Retinoid X receptor alpha) \| \| P20848 \| A1ATR_HUMAN \| Alpha-1-antitrypsin-related protein (AAT-related protein) (Protease inhibitor 1-like) (Serpin A2) \| \| P21399 \| ACOHC_HUMAN \| Cytoplasmic aconitate hydratase (Aconitase) (EC 4.2.1.3) (Citrate hydro-lyase) (Ferritin repressor protein) (Iron regulatory protein 1) (IRP1) (Iron-responsive element-binding protein 1) (IRE-BP 1) \| \| P21709 \| EPHA1_HUMAN \| Ephrin type-A receptor 1 (hEpha1) (EC 2.7.10.1) (EPH tyrosine kinase) (EPH tyrosine kinase 1) (Erythropoietin-producing hepatoma receptor) (Tyrosine-protein kinase receptor EPH) \| \| P21860 \| ERBB3_HUMAN \| Receptor tyrosine-protein kinase erbB-3 (EC 2.7.10.1) (Proto-oncogene-like protein c-ErbB-3) (Tyrosine kinase-type cell surface receptor HER3) \| \| P22059 \| OSBP1_HUMAN \| Oxysterol-binding protein 1 \| \| P22607 \| FGFR3_HUMAN \| Fibroblast growth factor receptor 3 (FGFR-3) (EC 2.7.10.1) (CD antigen CD333) \| \| P23458 \| JAK1_HUMAN \| Tyrosine-protein kinase JAK1 (EC 2.7.10.2) (Janus kinase 1) (JAK-1) \| \| P23528 \| COF1_HUMAN \| Cofilin-1 (18 kDa phosphoprotein) (p18) (Cofilin, non-muscle isoform) \| \| P23771 \| GATA3_HUMAN \| Trans-acting T-cell-specific transcription factor GATA-3 (GATA-binding factor 3) \| \| P24941 \| CDK2_HUMAN \| Cyclin-dependent kinase 2 (EC 2.7.11.22) (Cell division protein kinase 2) (p33 protein kinase) \| \| P25054 \| APC_HUMAN \| Adenomatous polyposis coli protein (Protein APC) (Deleted in polyposis 2.5) \| \| P25774 \| CATS_HUMAN \| Cathepsin S (EC 3.4.22.27) \| \| P25963 \| IKBA_HUMAN \| NF-kappa-B inhibitor alpha (I-kappa-B-alpha) (IkB-alpha) (IkappaBalpha) (Major histocompatibility complex enhancer-binding protein MAD3) \| \| P27361 \| MK03_HUMAN \| Mitogen-activated protein kinase 3 (MAP kinase 3) (MAPK 3) (EC 2.7.11.24) (ERT2) (Extracellular signal-regulated kinase 1) (ERK-1) (Insulin-stimulated MAP2 kinase) (MAP kinase isoform p44) (p44-MAPK) (Microtubule-associated protein 2 kinase) (p44-ERK1) \| \| P28329 \| CLAT_HUMAN \| Choline O-acetyltransferase (CHOACTase) (ChAT) (Choline acetylase) (EC 2.3.1.6) \| \| P28482 \| MK01_HUMAN \| Mitogen-activated protein kinase 1 (MAP kinase 1) (MAPK 1) (EC 2.7.11.24) (ERT1) (Extracellular signal-regulated kinase 2) (ERK-2) (MAP kinase isoform p42) (p42-MAPK) (Mitogen-activated protein kinase 2) (MAP kinase 2) (MAPK 2) \| \| P28562 \| DUS1_HUMAN \| Dual specificity protein phosphatase 1 (EC 3.1.3.16) (EC 3.1.3.48) (Dual specificity protein phosphatase hVH1) (Mitogen-activated protein kinase phosphatase 1) (MAP kinase phosphatase 1) (MKP-1) (Protein-tyrosine phosphatase CL100) \| \| P29323 \| EPHB2_HUMAN \| Ephrin type-B receptor 2 (EC 2.7.10.1) (Developmentally-regulated Eph-related tyrosine kinase) (ELK-related tyrosine kinase) (EPH tyrosine kinase 3) (EPH-like kinase 5) (EK5) (hEK5) (Renal carcinoma antigen NY-REN-47) (Tyrosine-protein kinase TYRO5) (Tyrosine-protein kinase receptor EPH-3) [Cleaved into: EphB2/CTF1; EphB2/CTF2] \| \| P29353 \| SHC1_HUMAN \| SHC-transforming protein 1 (SHC-transforming protein 3) (SHC-transforming protein A) (Src homology 2 domain-containing-transforming protein C1) (SH2 domain protein C1) \| \| P29597 \| TYK2_HUMAN \| Non-receptor tyrosine-protein kinase TYK2 (EC 2.7.10.2) \| \| P30304 \| MPIP1_HUMAN \| M-phase inducer phosphatase 1 (EC 3.1.3.48) (Dual specificity phosphatase Cdc25A) \| \| P32004 \| L1CAM_HUMAN \| Neural cell adhesion molecule L1 (N-CAM-L1) (NCAM-L1) (CD antigen CD171) \| \| P33993 \| MCM7_HUMAN \| DNA replication licensing factor MCM7 (EC 3.6.4.12) (CDC47 homolog) (P1.1-MCM3) \| \| P34972 \| CNR2_HUMAN \| Cannabinoid receptor 2 (CB-2) (CB2) (hCB2) (CX5) \| \| P35222 \| CTNB1_HUMAN \| Catenin beta-1 (Beta-catenin) \| \| P35568 \| IRS1_HUMAN \| Insulin receptor substrate 1 (IRS-1) \| \| P35637 \| FUS_HUMAN \| RNA-binding protein FUS (75 kDa DNA-pairing protein) (Oncogene FUS) (Oncogene TLS) (POMp75) (Translocated in liposarcoma protein) \| \| P35670 \| ATP7B_HUMAN \| Copper-transporting ATPase 2 (EC 7.2.2.8) (Copper pump 2) (Wilson disease-associated protein) [Cleaved into: WND/140 kDa] \| \| P35916 \| VGFR3_HUMAN \| Vascular endothelial growth factor receptor 3 (VEGFR-3) (EC 2.7.10.1) (Fms-like tyrosine kinase 4) (FLT-4) (Tyrosine-protein kinase receptor FLT4) \| \| P36507 \| MP2K2_HUMAN \| Dual specificity mitogen-activated protein kinase kinase 2 (MAP kinase kinase 2) (MAPKK 2) (EC 2.7.12.2) (ERK activator kinase 2) (MAPK/ERK kinase 2) (MEK 2) \| \| P37840 \| SYUA_HUMAN \| Alpha-synuclein (Non-A beta component of AD amyloid) (Non-A4 component of amyloid precursor) (NACP) \| \| P40238 \| TPOR_HUMAN \| Thrombopoietin receptor (TPO-R) (Myeloproliferative leukemia protein) (Proto-oncogene c-Mpl) (CD antigen CD110) \| \| P40763 \| STAT3_HUMAN \| Signal transducer and activator of transcription 3 (Acute-phase response factor) \| \| P41212 \| ETV6_HUMAN \| Transcription factor ETV6 (ETS translocation variant 6) (ETS-related protein Tel1) (Tel) \| \| P42224 \| STAT1_HUMAN \| Signal transducer and activator of transcription 1-alpha/beta (Transcription factor ISGF-3 components p91/p84) \| \| P42229 \| STA5A_HUMAN \| Signal transducer and activator of transcription 5A \| \| P42345 \| MTOR_HUMAN \| Serine/threonine-protein kinase mTOR (EC 2.7.11.1) (FK506-binding protein 12-rapamycin complex-associated protein 1) (FKBP12-rapamycin complex-associated protein) (Mammalian target of rapamycin) (mTOR) (Mechanistic target of rapamycin) (Rapamycin and FKBP12 target 1) (Rapamycin target protein 1) \| \| P42858 \| HD_HUMAN \| Huntingtin (Huntington disease protein) (HD protein) [Cleaved into: Huntingtin, myristoylated N-terminal fragment] \| \| P43243 \| MATR3_HUMAN \| Matrin-3 \| \| P43403 \| ZAP70_HUMAN \| Tyrosine-protein kinase ZAP-70 (EC 2.7.10.2) (70 kDa zeta-chain associated protein) (Syk-related tyrosine kinase) \| \| P45983 \| MK08_HUMAN \| Mitogen-activated protein kinase 8 (MAP kinase 8) (MAPK 8) (EC 2.7.11.24) (JNK-46) (Stress-activated protein kinase 1c) (SAPK1c) (Stress-activated protein kinase JNK1) (c-Jun N-terminal kinase 1) \| \| P45984 \| MK09_HUMAN \| Mitogen-activated protein kinase 9 (MAP kinase 9) (MAPK 9) (EC 2.7.11.24) (JNK-55) (Stress-activated protein kinase 1a) (SAPK1a) (Stress-activated protein kinase JNK2) (c-Jun N-terminal kinase 2) \| \| P46734 \| MP2K3_HUMAN \| Dual specificity mitogen-activated protein kinase kinase 3 (MAP kinase kinase 3) (MAPKK 3) (EC 2.7.12.2) (MAPK/ERK kinase 3) (MEK 3) (Stress-activated protein kinase kinase 2) (SAPK kinase 2) (SAPKK-2) (SAPKK2) \| \| P46937 \| YAP1_HUMAN \| Transcriptional coactivator YAP1 (Yes-associated protein 1) (Protein yorkie homolog) (Yes-associated protein YAP65 homolog) \| \| P48436 \| SOX9_HUMAN \| Transcription factor SOX-9 \| \| P49137 \| MAPK2_HUMAN \| MAP kinase-activated protein kinase 2 (MAPK-activated protein kinase 2) (MAPKAP kinase 2) (MAPKAP-K2) (MAPKAPK-2) (MK-2) (MK2) (EC 2.7.11.1) \| \| P49736 \| MCM2_HUMAN \| DNA replication licensing factor MCM2 (EC 3.6.4.12) (Minichromosome maintenance protein 2 homolog) (Nuclear protein BM28) \| \| P50750 \| CDK9_HUMAN \| Cyclin-dependent kinase 9 (EC 2.7.11.22) (EC 2.7.11.23) (C-2K) (Cell division cycle 2-like protein kinase 4) (Cell division protein kinase 9) (Serine/threonine-protein kinase PITALRE) (Tat-associated kinase complex catalytic subunit) \| \| P50995 \| ANX11_HUMAN \| Annexin A11 (56 kDa autoantigen) (Annexin XI) (Annexin-11) (Calcyclin-associated annexin 50) (CAP-50) \| \| P51452 \| DUS3_HUMAN \| Dual specificity protein phosphatase 3 (EC 3.1.3.16) (EC 3.1.3.48) (Dual specificity protein phosphatase VHR) (Vaccinia H1-related phosphatase) (VHR) \| \| P51955 \| NEK2_HUMAN \| Serine/threonine-protein kinase Nek2 (EC 2.7.11.1) (HSPK 21) (Never in mitosis A-related kinase 2) (NimA-related protein kinase 2) (NimA-like protein kinase 1) \| \| P52333 \| JAK3_HUMAN \| Tyrosine-protein kinase JAK3 (EC 2.7.10.2) (Janus kinase 3) (JAK-3) (Leukocyte janus kinase) (L-JAK) \| \| P52564 \| MP2K6_HUMAN \| Dual specificity mitogen-activated protein kinase kinase 6 (MAP kinase kinase 6) (MAPKK 6) (EC 2.7.12.2) (MAPK/ERK kinase 6) (MEK 6) (Stress-activated protein kinase kinase 3) (SAPK kinase 3) (SAPKK-3) (SAPKK3) \| \| P53350 \| PLK1_HUMAN \| Serine/threonine-protein kinase PLK1 (EC 2.7.11.21) (Polo-like kinase 1) (PLK-1) (Serine/threonine-protein kinase 13) (STPK13) \| \| P53355 \| DAPK1_HUMAN \| Death-associated protein kinase 1 (DAP kinase 1) (EC 2.7.11.1) \| \| P53779 \| MK10_HUMAN \| Mitogen-activated protein kinase 10 (MAP kinase 10) (MAPK 10) (EC 2.7.11.24) (MAP kinase p49 3F12) (Stress-activated protein kinase 1b) (SAPK1b) (Stress-activated protein kinase JNK3) (c-Jun N-terminal kinase 3) \| \| P54646 \| AAPK2_HUMAN \| 5'-AMP-activated protein kinase catalytic subunit alpha-2 (AMPK subunit alpha-2) (EC 2.7.11.1) (Acetyl-CoA carboxylase kinase) (ACACA kinase) (Hydroxymethylglutaryl-CoA reductase kinase) (HMGCR kinase) (EC 2.7.11.31) \| \| P54760 \| EPHB4_HUMAN \| Ephrin type-B receptor 4 (EC 2.7.10.1) (Hepatoma transmembrane kinase) (Tyrosine-protein kinase TYRO11) \| \| P54764 \| EPHA4_HUMAN \| Ephrin type-A receptor 4 (EC 2.7.10.1) (EPH-like kinase 8) (EK8) (hEK8) (Tyrosine-protein kinase TYRO1) (Tyrosine-protein kinase receptor SEK) \| \| P55017 \| S12A3_HUMAN \| Solute carrier family 12 member 3 (Na-Cl cotransporter) (NCC) (Na-Cl symporter) (Thiazide-sensitive sodium-chloride cotransporter) \| \| P61764 \| STXB1_HUMAN \| Syntaxin-binding protein 1 (MUNC18-1) (N-Sec1) (Protein unc-18 homolog 1) (Unc18-1) (Protein unc-18 homolog A) (Unc-18A) (p67) \| \| P62714 \| PP2AB_HUMAN \| Serine/threonine-protein phosphatase 2A catalytic subunit beta isoform (PP2A-beta) (EC 3.1.3.16) \| \| P63010 \| AP2B1_HUMAN \| AP-2 complex subunit beta (AP105B) (Adaptor protein complex AP-2 subunit beta) (Adaptor-related protein complex 2 subunit beta) (Beta-2-adaptin) (Beta-adaptin) (Clathrin assembly protein complex 2 beta large chain) (Plasma membrane adaptor HA2/AP2 adaptin beta subunit) \| \| P68104 \| EF1A1_HUMAN \| Elongation factor 1-alpha 1 (EF-1-alpha-1) (EC 3.6.5.-) (Elongation factor Tu) (EF-Tu) (Eukaryotic elongation factor 1 A-1) (eEF1A-1) (Leukocyte receptor cluster member 7) \| \| P68431 \| H31_HUMAN \| Histone H3.1 (Histone H3/a) (Histone H3/b) (Histone H3/c) (Histone H3/d) (Histone H3/f) (Histone H3/h) (Histone H3/i) (Histone H3/j) (Histone H3/k) (Histone H3/l) \| \| P78362 \| SRPK2_HUMAN \| SRSF protein kinase 2 (EC 2.7.11.1) (SFRS protein kinase 2) (Serine/arginine-rich protein-specific kinase 2) (SR-protein-specific kinase 2) [Cleaved into: SRSF protein kinase 2 N-terminal; SRSF protein kinase 2 C-terminal] \| \| P85037 \| FOXK1_HUMAN \| Forkhead box protein K1 (Myocyte nuclear factor) (MNF) \| \| Q00536 \| CDK16_HUMAN \| Cyclin-dependent kinase 16 (EC 2.7.11.22) (Cell division protein kinase 16) (PCTAIRE-motif protein kinase 1) (Serine/threonine-protein kinase PCTAIRE-1) \| \| Q00613 \| HSF1_HUMAN \| Heat shock factor protein 1 (HSF 1) (Heat shock transcription factor 1) (HSTF 1) \| \| Q01196 \| RUNX1_HUMAN \| Runt-related transcription factor 1 (Acute myeloid leukemia 1 protein) (Core-binding factor subunit alpha-2) (CBF-alpha-2) (Oncogene AML-1) (Polyomavirus enhancer-binding protein 2 alpha B subunit) (PEA2-alpha B) (PEBP2-alpha B) (SL3-3 enhancer factor 1 alpha B subunit) (SL3/AKV core-binding factor alpha B subunit) \| \| Q01581 \| HMCS1_HUMAN \| Hydroxymethylglutaryl-CoA synthase, cytoplasmic (HMG-CoA synthase) (EC 2.3.3.10) (3-hydroxy-3-methylglutaryl coenzyme A synthase) \| \| Q01813 \| PFKAP_HUMAN \| ATP-dependent 6-phosphofructokinase, platelet type (ATP-PFK) (PFK-P) (EC 2.7.1.11) (6-phosphofructokinase type C) (Phosphofructo-1-kinase isozyme C) (PFK-C) (Phosphohexokinase) \| \| Q02750 \| MP2K1_HUMAN \| Dual specificity mitogen-activated protein kinase kinase 1 (MAP kinase kinase 1) (MAPKK 1) (MKK1) (EC 2.7.12.2) (ERK activator kinase 1) (MAPK/ERK kinase 1) (MEK 1) \| \| Q02763 \| TIE2_HUMAN \| Angiopoietin-1 receptor (EC 2.7.10.1) (Endothelial tyrosine kinase) (Tunica interna endothelial cell kinase) (Tyrosine kinase with Ig and EGF homology domains-2) (Tyrosine-protein kinase receptor TEK) (Tyrosine-protein kinase receptor TIE-2) (hTIE2) (p140 TEK) (CD antigen CD202b) \| \| Q02952 \| AKA12_HUMAN \| A-kinase anchor protein 12 (AKAP-12) (A-kinase anchor protein 250 kDa) (AKAP 250) (Gravin) (Myasthenia gravis autoantigen) \| \| Q04637 \| IF4G1_HUMAN \| Eukaryotic translation initiation factor 4 gamma 1 (eIF-4-gamma 1) (eIF-4G 1) (eIF-4G1) (p220) \| \| Q05655 \| KPCD_HUMAN \| Protein kinase C delta type (EC 2.7.11.13) (Tyrosine-protein kinase PRKCD) (EC 2.7.10.2) (nPKC-delta) [Cleaved into: Protein kinase C delta type regulatory subunit; Protein kinase C delta type catalytic subunit (Sphingosine-dependent protein kinase-1) (SDK1)] \| \| Q05923 \| DUS2_HUMAN \| Dual specificity protein phosphatase 2 (EC 3.1.3.16) (EC 3.1.3.48) (Dual specificity protein phosphatase PAC-1) \| \| Q06124 \| PTN11_HUMAN \| Tyrosine-protein phosphatase non-receptor type 11 (EC 3.1.3.48) (Protein-tyrosine phosphatase 1D) (PTP-1D) (Protein-tyrosine phosphatase 2C) (PTP-2C) (SH-PTP2) (SHP-2) (Shp2) (SH-PTP3) \| \| Q09428 \| ABCC8_HUMAN \| ATP-binding cassette sub-family C member 8 (Sulfonylurea receptor 1) \| \| Q12778 \| FOXO1_HUMAN \| Forkhead box protein O1 (Forkhead box protein O1A) (Forkhead in rhabdomyosarcoma) \| \| Q12988 \| HSPB3_HUMAN \| Heat shock protein beta-3 (HspB3) (Heat shock 17 kDa protein) (HSP 17) (Protein 3) \| \| Q13163 \| MP2K5_HUMAN \| Dual specificity mitogen-activated protein kinase kinase 5 (MAP kinase kinase 5) (MAPKK 5) (EC 2.7.12.2) (MAPK/ERK kinase 5) (MEK 5) \| \| Q13177 \| PAK2_HUMAN \| Serine/threonine-protein kinase PAK 2 (EC 2.7.11.1) (Gamma-PAK) (PAK65) (S6/H4 kinase) (p21-activated kinase 2) (PAK-2) (p58) [Cleaved into: PAK-2p27 (p27); PAK-2p34 (p34) (C-t-PAK2)] \| \| Q13263 \| TIF1B_HUMAN \| Transcription intermediary factor 1-beta (TIF1-beta) (E3 SUMO-protein ligase TRIM28) (EC 2.3.2.27) (KRAB-associated protein 1) (KAP-1) (KRAB-interacting protein 1) (KRIP-1) (Nuclear corepressor KAP-1) (RING finger protein 96) (RING-type E3 ubiquitin transferase TIF1-beta) (Tripartite motif-containing protein 28) \| \| Q13470 \| TNK1_HUMAN \| Non-receptor tyrosine-protein kinase TNK1 (EC 2.7.10.2) (CD38 negative kinase 1) \| \| Q13501 \| SQSTM_HUMAN \| Sequestosome-1 (EBI3-associated protein of 60 kDa) (EBIAP) (p60) (Phosphotyrosine-independent ligand for the Lck SH2 domain of 62 kDa) (Ubiquitin-binding protein p62) \| \| Q13522 \| PPR1A_HUMAN \| Protein phosphatase 1 regulatory subunit 1A (Protein phosphatase inhibitor 1) (I-1) (IPP-1) \| \| Q13523 \| PRP4B_HUMAN \| Serine/threonine-protein kinase PRP4 homolog (EC 2.7.11.1) (PRP4 kinase) (PRP4 pre-mRNA-processing factor 4 homolog) \| \| Q13535 \| ATR_HUMAN \| Serine/threonine-protein kinase ATR (EC 2.7.11.1) (Ataxia telangiectasia and Rad3-related protein) (FRAP-related protein 1) \| \| Q14118 \| DAG1_HUMAN \| Dystroglycan 1 (Dystroglycan) (Dystrophin-associated glycoprotein 1) [Cleaved into: Alpha-dystroglycan (Alpha-DG); Beta-dystroglycan (Beta-DG)] \| \| Q14203 \| DCTN1_HUMAN \| Dynactin subunit 1 (150 kDa dynein-associated polypeptide) (DAP-150) (DP-150) (p135) (p150-glued) \| \| Q14653 \| IRF3_HUMAN \| Interferon regulatory factor 3 (IRF-3) \| \| Q14934 \| NFAC4_HUMAN \| Nuclear factor of activated T-cells, cytoplasmic 4 (NF-ATc4) (NFATc4) (T-cell transcription factor NFAT3) (NF-AT3) \| \| Q15118 \| PDK1_HUMAN \| [Pyruvate dehydrogenase (acetyl-transferring)] kinase isozyme 1, mitochondrial (EC 2.7.11.2) (Pyruvate dehydrogenase kinase isoform 1) (PDH kinase 1) \| \| Q15120 \| PDK3_HUMAN \| [Pyruvate dehydrogenase (acetyl-transferring)] kinase isozyme 3, mitochondrial (EC 2.7.11.2) (Pyruvate dehydrogenase kinase isoform 3) \| \| Q15303 \| ERBB4_HUMAN \| Receptor tyrosine-protein kinase erbB-4 (EC 2.7.10.1) (Proto-oncogene-like protein c-ErbB-4) (Tyrosine kinase-type cell surface receptor HER4) (p180erbB4) [Cleaved into: ERBB4 intracellular domain (4ICD) (E4ICD) (s80HER4)] \| \| Q15418 \| KS6A1_HUMAN \| Ribosomal protein S6 kinase alpha-1 (S6K-alpha-1) (EC 2.7.11.1) (90 kDa ribosomal protein S6 kinase 1) (p90-RSK 1) (p90RSK1) (p90S6K) (MAP kinase-activated protein kinase 1a) (MAPK-activated protein kinase 1a) (MAPKAP kinase 1a) (MAPKAPK-1a) (Ribosomal S6 kinase 1) (RSK-1) \| \| Q15722 \| LT4R1_HUMAN \| Leukotriene B4 receptor 1 (LTB4-R 1) (LTB4-R1) (Chemoattractant receptor-like 1) (G-protein coupled receptor 16) (P2Y purinoceptor 7) (P2Y7) \| \| Q15831 \| STK11_HUMAN \| Serine/threonine-protein kinase STK11 (EC 2.7.11.1) (Liver kinase B1) (LKB1) (hLKB1) (Renal carcinoma antigen NY-REN-19) \| \| Q16236 \| NF2L2_HUMAN \| Nuclear factor erythroid 2-related factor 2 (NF-E2-related factor 2) (NFE2-related factor 2) (Nrf-2) (HEBP1) (Nuclear factor, erythroid derived 2, like 2) \| \| Q16513 \| PKN2_HUMAN \| Serine/threonine-protein kinase N2 (EC 2.7.11.13) (PKN gamma) (Protein kinase C-like 2) (Protein-kinase C-related kinase 2) \| \| Q16555 \| DPYL2_HUMAN \| Dihydropyrimidinase-related protein 2 (DRP-2) (Collapsin response mediator protein 2) (CRMP-2) (N2A3) (Unc-33-like phosphoprotein 2) (ULIP-2) \| \| Q16566 \| KCC4_HUMAN \| Calcium/calmodulin-dependent protein kinase type IV (CaMK IV) (EC 2.7.11.17) (CaM kinase-GR) \| \| Q16620 \| NTRK2_HUMAN \| BDNF/NT-3 growth factors receptor (EC 2.7.10.1) (GP145-TrkB) (Trk-B) (Neurotrophic tyrosine kinase receptor type 2) (TrkB tyrosine kinase) (Tropomyosin-related kinase B) \| \| Q16665 \| HIF1A_HUMAN \| Hypoxia-inducible factor 1-alpha (HIF-1-alpha) (HIF1-alpha) (ARNT-interacting protein) (Basic-helix-loop-helix-PAS protein MOP1) (Class E basic helix-loop-helix protein 78) (bHLHe78) (Member of PAS protein 1) (PAS domain-containing protein 8) \| \| Q16828 \| DUS6_HUMAN \| Dual specificity protein phosphatase 6 (EC 3.1.3.16) (EC 3.1.3.48) (Dual specificity protein phosphatase PYST1) (Mitogen-activated protein kinase phosphatase 3) (MAP kinase phosphatase 3) (MKP-3) \| \| Q16829 \| DUS7_HUMAN \| Dual specificity protein phosphatase 7 (EC 3.1.3.16) (EC 3.1.3.48) (Dual specificity protein phosphatase PYST2) \| \| Q16832 \| DDR2_HUMAN \| Discoidin domain-containing receptor 2 (Discoidin domain receptor 2) (EC 2.7.10.1) (CD167 antigen-like family member B) (Discoidin domain-containing receptor tyrosine kinase 2) (Neurotrophic tyrosine kinase, receptor-related 3) (Receptor protein-tyrosine kinase TKT) (Tyrosine-protein kinase TYRO10) (CD antigen CD167b) \| \| Q18PE1 \| DOK7_HUMAN \| Protein Dok-7 (Downstream of tyrosine kinase 7) \| \| Q56UN5 \| M3K19_HUMAN \| Mitogen-activated protein kinase kinase kinase 19 (EC 2.7.11.1) (Regulated in COPD, protein kinase) (SPS1/STE20-related protein kinase YSK4) \| \| Q5S007 \| LRRK2_HUMAN \| Leucine-rich repeat serine/threonine-protein kinase 2 (EC 2.7.11.1) (EC 3.6.5.-) (Dardarin) \| \| Q5TCY1 \| TTBK1_HUMAN \| Tau-tubulin kinase 1 (EC 2.7.11.1) (Brain-derived tau kinase) \| \| Q5VST9 \| OBSCN_HUMAN \| Obscurin (EC 2.7.11.1) (Obscurin-RhoGEF) (Obscurin-myosin light chain kinase) (Obscurin-MLCK) \| \| Q86SF2 \| GALT7_HUMAN \| N-acetylgalactosaminyltransferase 7 (EC 2.4.1.41) (Polypeptide GalNAc transferase 7) (GalNAc-T7) (pp-GaNTase 7) (Protein-UDP acetylgalactosaminyltransferase 7) (UDP-GalNAc:polypeptide N-acetylgalactosaminyltransferase 7) \| \| Q86W92 \| LIPB1_HUMAN \| Liprin-beta-1 (Protein tyrosine phosphatase receptor type f polypeptide-interacting protein-binding protein 1) (PTPRF-interacting protein-binding protein 1) (hSGT2) \| \| Q86YV5 \| PRAG1_HUMAN \| Inactive tyrosine-protein kinase PRAG1 (PEAK1-related kinase-activating pseudokinase 1) (Pragmin) (Sugen kinase 223) (SgK223) \| \| Q8IVT5 \| KSR1_HUMAN \| Kinase suppressor of Ras 1 (EC 2.7.11.1) \| \| Q8N3U4 \| STAG2_HUMAN \| Cohesin subunit SA-2 (SCC3 homolog 2) (Stromal antigen 2) \| \| Q8NEZ4 \| KMT2C_HUMAN \| Histone-lysine N-methyltransferase 2C (Lysine N-methyltransferase 2C) (EC 2.1.1.364) (Homologous to ALR protein) (Myeloid/lymphoid or mixed-lineage leukemia protein 3) \| \| Q92574 \| TSC1_HUMAN \| Hamartin (Tuberous sclerosis 1 protein) \| \| Q92772 \| CDKL2_HUMAN \| Cyclin-dependent kinase-like 2 (EC 2.7.11.22) (Protein kinase p56 KKIAMRE) (Serine/threonine-protein kinase KKIAMRE) \| \| Q92835 \| SHIP1_HUMAN \| Phosphatidylinositol 3,4,5-trisphosphate 5-phosphatase 1 (EC 3.1.3.86) (Inositol polyphosphate-5-phosphatase D) (EC 3.1.3.56) (Inositol polyphosphate-5-phosphatase of 145 kDa) (SIP-145) (Phosphatidylinositol 4,5-bisphosphate 5-phosphatase) (EC 3.1.3.36) (SH2 domain-containing inositol 5'-phosphatase 1) (SH2 domain-containing inositol phosphatase 1) (SHIP-1) (p150Ship) (hp51CN) \| \| Q96C90 \| PP14B_HUMAN \| Protein phosphatase 1 regulatory subunit 14B (Phospholipase C-beta-3 neighbouring gene protein) \| \| Q96CV9 \| OPTN_HUMAN \| Optineurin (E3-14.7K-interacting protein) (FIP-2) (Huntingtin yeast partner L) (Huntingtin-interacting protein 7) (HIP-7) (Huntingtin-interacting protein L) (NEMO-related protein) (Optic neuropathy-inducing protein) (Transcription factor IIIA-interacting protein) (TFIIIA-IntP) \| \| Q96HC4 \| PDLI5_HUMAN \| PDZ and LIM domain protein 5 (Enigma homolog) (Enigma-like PDZ and LIM domains protein) \| \| Q96J92 \| WNK4_HUMAN \| Serine/threonine-protein kinase WNK4 (EC 2.7.11.1) (Protein kinase lysine-deficient 4) (Protein kinase with no lysine 4) \| \| Q96Q42 \| ALS2_HUMAN \| Alsin (Amyotrophic lateral sclerosis 2 chromosomal region candidate gene 6 protein) (Amyotrophic lateral sclerosis 2 protein) \| \| Q96QT4 \| TRPM7_HUMAN \| Transient receptor potential cation channel subfamily M member 7 (EC 2.7.11.1) (Channel-kinase 1) (Long transient receptor potential channel 7) (LTrpC-7) (LTrpC7) \| \| Q96ST2 \| IWS1_HUMAN \| Protein IWS1 homolog (IWS1-like protein) \| \| Q96ST3 \| SIN3A_HUMAN \| Paired amphipathic helix protein Sin3a (Histone deacetylase complex subunit Sin3a) (Transcriptional corepressor Sin3a) \| \| Q99558 \| M3K14_HUMAN \| Mitogen-activated protein kinase kinase kinase 14 (EC 2.7.11.25) (NF-kappa-beta-inducing kinase) (HsNIK) (Serine/threonine-protein kinase NIK) \| \| Q9BVS4 \| RIOK2_HUMAN \| Serine/threonine-protein kinase RIO2 (EC 2.7.11.1) (RIO kinase 2) \| \| Q9BYW2 \| SETD2_HUMAN \| Histone-lysine N-methyltransferase SETD2 (EC 2.1.1.359) (HIF-1) (Huntingtin yeast partner B) (Huntingtin-interacting protein 1) (HIP-1) (Huntingtin-interacting protein B) (Lysine N-methyltransferase 3A) (Protein-lysine N-methyltransferase SETD2) (EC 2.1.1.-) (SET domain-containing protein 2) (hSET2) (p231HBP) \| \| Q9H0K1 \| SIK2_HUMAN \| Serine/threonine-protein kinase SIK2 (EC 2.7.11.1) (Qin-induced kinase) (Salt-inducible kinase 2) (SIK-2) (Serine/threonine-protein kinase SNF1-like kinase 2) \| \| Q9H6X2 \| ANTR1_HUMAN \| Anthrax toxin receptor 1 (Tumor endothelial marker 8) \| \| Q9H902 \| REEP1_HUMAN \| Receptor expression-enhancing protein 1 (Spastic paraplegia 31 protein) \| \| Q9HBH9 \| MKNK2_HUMAN \| MAP kinase-interacting serine/threonine-protein kinase 2 (EC 2.7.11.1) (MAP kinase signal-integrating kinase 2) (MAPK signal-integrating kinase 2) (Mnk2) \| \| Q9HC35 \| EMAL4_HUMAN \| Echinoderm microtubule-associated protein-like 4 (EMAP-4) (Restrictedly overexpressed proliferation-associated protein) (Ropp 120) \| \| Q9NWH9 \| SLTM_HUMAN \| SAFB-like transcription modulator (Modulator of estrogen-induced transcription) \| \| Q9NYF8 \| BCLF1_HUMAN \| Bcl-2-associated transcription factor 1 (Btf) (BCLAF1 and THRAP3 family member 1) \| \| Q9NYV4 \| CDK12_HUMAN \| Cyclin-dependent kinase 12 (EC 2.7.11.22) (EC 2.7.11.23) (Cdc2-related kinase, arginine/serine-rich) (CrkRS) (Cell division cycle 2-related protein kinase 7) (CDC2-related protein kinase 7) (Cell division protein kinase 12) (hCDK12) \| \| Q9NZL4 \| HPBP1_HUMAN \| Hsp70-binding protein 1 (HspBP1) (Heat shock protein-binding protein 1) (Hsp70-binding protein 2) (HspBP2) (Hsp70-interacting protein 1) (Hsp70-interacting protein 2) \| \| Q9P1W9 \| PIM2_HUMAN \| Serine/threonine-protein kinase pim-2 (EC 2.7.11.1) (Pim-2h) \| \| Q9P2K8 \| E2AK4_HUMAN \| eIF-2-alpha kinase GCN2 (EC 2.7.11.1) (Eukaryotic translation initiation factor 2-alpha kinase 4) (GCN2-like protein) \| \| Q9P2Y5 \| UVRAG_HUMAN \| UV radiation resistance-associated gene protein (p63) \| \| Q9UHD2 \| TBK1_HUMAN \| Serine/threonine-protein kinase TBK1 (EC 2.7.11.1) (NF-kappa-B-activating kinase) (T2K) (TANK-binding kinase 1) \| \| Q9UHD9 \| UBQL2_HUMAN \| Ubiquilin-2 (Chap1) (DSK2 homolog) (Protein linking IAP with cytoskeleton 2) (PLIC-2) (hPLIC-2) (Ubiquitin-like product Chap1/Dsk2) \| \| Q9UJU2 \| LEF1_HUMAN \| Lymphoid enhancer-binding factor 1 (LEF-1) (T cell-specific transcription factor 1-alpha) (TCF1-alpha) \| \| Q9UK32 \| KS6A6_HUMAN \| Ribosomal protein S6 kinase alpha-6 (S6K-alpha-6) (EC 2.7.11.1) (90 kDa ribosomal protein S6 kinase 6) (p90-RSK 6) (p90RSK6) (Ribosomal S6 kinase 4) (RSK-4) (pp90RSK4) \| \| Q9UPN9 \| TRI33_HUMAN \| E3 ubiquitin-protein ligase TRIM33 (EC 2.3.2.27) (Ectodermin homolog) (RET-fused gene 7 protein) (Protein Rfg7) (RING-type E3 ubiquitin transferase TRIM33) (Transcription intermediary factor 1-gamma) (TIF1-gamma) (Tripartite motif-containing protein 33) \| \| Q9UPX8 \| SHAN2_HUMAN \| SH3 and multiple ankyrin repeat domains protein 2 (Shank2) (Cortactin-binding protein 1) (CortBP1) (Proline-rich synapse-associated protein 1) \| \| Q9UQM7 \| KCC2A_HUMAN \| Calcium/calmodulin-dependent protein kinase type II subunit alpha (CaM kinase II subunit alpha) (CaMK-II subunit alpha) (EC 2.7.11.17) \| \| Q9Y2U5 \| M3K2_HUMAN \| Mitogen-activated protein kinase kinase kinase 2 (EC 2.7.11.25) (MAPK/ERK kinase kinase 2) (MEK kinase 2) (MEKK 2) \| \| Q9Y2W1 \| TR150_HUMAN \| Thyroid hormone receptor-associated protein 3 (BCLAF1 and THRAP3 family member 2) (Thyroid hormone receptor-associated protein complex 150 kDa component) (Trap150) \| \| Q9Y478 \| AAKB1_HUMAN \| 5'-AMP-activated protein kinase subunit beta-1 (AMPK subunit beta-1) (AMPKb) \| \| Q9Y4L1 \| HYOU1_HUMAN \| Hypoxia up-regulated protein 1 (150 kDa oxygen-regulated protein) (ORP-150) (170 kDa glucose-regulated protein) (GRP-170) \| \| Q9Y6E0 \| STK24_HUMAN \| Serine/threonine-protein kinase 24 (EC 2.7.11.1) (Mammalian STE20-like protein kinase 3) (MST-3) (STE20-like kinase MST3) [Cleaved into: Serine/threonine-protein kinase 24 36 kDa subunit (Mammalian STE20-like protein kinase 3 N-terminal) (MST3/N); Serine/threonine-protein kinase 24 12 kDa subunit (Mammalian STE20-like protein kinase 3 C-terminal) (MST3/C)] \| |
| --- | --- | --- | --- | --- | --- | --- | --- | --- | --- | --- | --- | --- | --- | --- | --- | --- | --- | --- | --- | --- | --- | --- | --- | --- | --- | --- | --- | --- | --- | --- | --- | --- | --- | --- | --- | --- | --- | --- | --- | --- | --- | --- | --- | --- | --- | --- | --- | --- | --- | --- | --- | --- | --- | --- | --- | --- | --- | --- | --- | --- | --- | --- | --- | --- | --- | --- | --- | --- | --- | --- | --- | --- | --- | --- | --- | --- | --- | --- | --- | --- | --- | --- | --- | --- | --- | --- | --- | --- | --- | --- | --- | --- | --- | --- | --- | --- | --- | --- | --- | --- | --- | --- | --- | --- | --- | --- | --- | --- | --- | --- | --- | --- | --- | --- | --- | --- | --- | --- | --- | --- | --- | --- | --- | --- | --- | --- | --- | --- | --- | --- | --- | --- | --- | --- | --- | --- | --- | --- | --- | --- | --- | --- | --- | --- | --- | --- | --- | --- | --- | --- | --- | --- | --- | --- | --- | --- | --- | --- | --- | --- | --- | --- | --- | --- | --- | --- | --- | --- | --- | --- | --- | --- | --- | --- | --- | --- | --- | --- | --- | --- | --- | --- | --- | --- | --- | --- | --- | --- | --- | --- | --- | --- | --- | --- | --- | --- | --- | --- | --- | --- | --- | --- | --- | --- | --- | --- | --- | --- | --- | --- | --- | --- | --- | --- | --- | --- | --- | --- | --- | --- | --- | --- | --- | --- | --- | --- | --- | --- | --- | --- | --- | --- | --- | --- | --- | --- | --- | --- | --- | --- | --- | --- | --- | --- | --- | --- | --- | --- | --- | --- | --- | --- | --- | --- | --- | --- | --- | --- | --- | --- | --- | --- | --- | --- | --- | --- | --- | --- | --- | --- | --- | --- | --- | --- | --- | --- | --- | --- | --- | --- | --- | --- | --- | --- | --- | --- | --- | --- | --- | --- | --- | --- | --- | --- | --- | --- | --- | --- | --- | --- | --- | --- | --- | --- | --- | --- | --- | --- | --- | --- | --- | --- | --- | --- | --- | --- | --- | --- | --- | --- | --- | --- | --- | --- | --- | --- | --- | --- | --- | --- | --- | --- | --- | --- | --- | --- | --- | --- | --- | --- | --- | --- | --- | --- | --- | --- | --- | --- | --- | --- | --- | --- | --- | --- | --- | --- | --- | --- | --- | --- | --- | --- | --- | --- | --- | --- | --- | --- | --- | --- | --- | --- | --- | --- | --- | --- | --- | --- | --- | --- | --- | --- | --- | --- | --- | --- | --- | --- | --- | --- | --- | --- | --- | --- | --- | --- | --- | --- | --- | --- | --- | --- | --- | --- | --- | --- | --- | --- | --- | --- | --- | --- | --- | --- | --- | --- | --- | --- | --- | --- | --- | --- | --- | --- | --- | --- | --- | --- | --- | --- | --- | --- | --- | --- | --- | --- | --- | --- | --- | --- | --- | --- | --- | --- | --- | --- | --- | --- | --- | --- | --- | --- | --- | --- | --- | --- | --- | --- | --- | --- | --- | --- | --- | --- | --- | --- | --- | --- | --- | --- | --- | --- | --- | --- | --- | --- | --- | --- | --- | --- | --- | --- | --- | --- | --- | --- | --- | --- | --- | --- | --- | --- | --- | --- | --- | --- | --- | --- | --- | --- | --- | --- | --- | --- | --- | --- | --- | --- | --- | --- | --- | --- | --- | --- | --- | --- | --- | --- | --- | --- | --- | --- | --- | --- | --- | --- | --- | --- | --- | --- | --- | --- | --- | --- | --- | --- | --- | --- | --- | --- | --- | --- | --- | --- | --- | --- | --- | --- | --- | --- | --- | --- | --- | --- | --- | --- | --- | --- | --- | --- | --- | --- | --- | --- | --- | --- | --- | --- | --- | --- | --- | --- | --- | --- | --- | --- | --- | --- | --- | --- | --- | --- | --- | --- | --- | --- | --- | --- | --- | --- | --- | --- | --- | --- | --- | --- | --- | --- | --- | --- | --- | --- | --- | --- | --- | --- | --- | --- | --- | --- | --- | --- | --- | --- | --- | --- | --- | --- | --- | --- | --- | --- | --- | --- | --- | --- | --- | --- | --- | --- | --- | --- | --- | --- | --- | --- | --- | --- | --- | --- | --- | --- | --- | --- | --- |
